# Artificial intelligence in autoimmune diseases: a bibliometric exploration of the past two decades

**DOI:** 10.3389/fimmu.2025.1525462

**Published:** 2025-04-22

**Authors:** Sidi Liu, Yang Liu, Ming Li, Shuangshuang Shang, Yunxiang Cao, Xi Shen, Chuanbing Huang

**Affiliations:** ^1^ Department of Rheumatology and Immunology, The First Affiliated Hospital of Anhui University of Traditional Chinese Medicine, Hefei, Anhui, China; ^2^ Center for Xin’an Medicine and Modernization of Traditional Chinese Medicine of Institute of Health and Medicine (IHM), The First Affiliated Hospital of Anhui University of Traditional Chinese Medicine, Hefei, Anhui, China; ^3^ Department of Orthopedics, The First Affiliated Hospital of Anhui University of Traditional Chinese Medicine, Hefei, Anhui, China

**Keywords:** artificial intelligence, autoimmune diseases, bibliometric exploration, forefront, content analysis

## Abstract

**Objective:**

Autoimmune diseases have long been recognized for their intricate nature and elusive mechanisms, presenting significant challenges in both diagnosis and treatment. The advent of artificial intelligence technology has opened up new possibilities for understanding, diagnosing, predicting, and managing autoimmune disorders. This study aims to explore the current state and emerging trends in the field through bibliometric analysis, providing guidance for future research directions.

**Methods:**

The study employed the Web of Science Core Collection database for data acquisition and performed bibliometric analysis using CiteSpace, HistCite Pro, and VOSviewer.

**Results:**

Over the past two decades, 1,695 publications emerged in this research field, including 1,409 research articles and 286 reviews. This investigation unveils the global development landscape predominantly led by the United States and China. The research identifies key institutions, such as Brigham & Women’s Hospital, influential journals like the Annals of the Rheumatic Diseases, distinguished authors including Katherine P. Liao, and pivotal articles. It visually maps out the research clusters’ evolutionary path over time and explores their applications in patient identification, risk factors, prognosis assessment, diagnosis, classification of disease subtypes, monitoring and decision support, and drug discovery.

**Conclusion:**

AI is increasingly recognized for its potential in the field of autoimmune diseases, yet it continues to face numerous challenges, including insufficient model validation and difficulties in data integration and computational power. Significant advancements have been demanded to enhance diagnostic precision, improve treatment methodologies, and establish robust frameworks for data protection, thereby facilitating more effective management of these complex conditions.

## 1 Introduction

Autoimmune diseases (AID) encompass a spectrum of conditions instigated by an anomalous immune reaction to internal antigens (1, 2). The intricacies and fundamental mechanisms of these disorders pose challenges in diagnosis and treatment. Although substantial headway has been achieved in comprehending the pathophysiology of AID in recent years, there remains a need for more refined diagnostic modalities and efficacious therapeutic approaches. The advent of artificial intelligence (AI) technology offers promising new avenues for advancing our understanding, diagnosis, and management of AID. AI algorithms have the capacity to scrutinize copious datasets from diverse origins such as electronic health records, laboratory indicators, medical imaging, and genomic information (3–5). Through discerning subtle patterns and associations, AI facilitates disease prognosis, early detection, and refinement of treatment modalities (5–8). Despite having demonstrated significant potential in the field of AID, AI has encountered numerous challenges related to models, data, and treatment. Future advancements are expected to be driven by data integration and algorithm optimization, aimed at enhancing diagnosis, treatment, and monitoring capabilities. The integration of AI in AID research has proliferated in tandem with advancements in AI technology in the last two decades. The objective of our investigation is to explore the evolution of this field over the past twenty years through a bibliometric lens.

Bibliometrics is a method that reveals the research patterns and trends in a specific field through quantitative and qualitative analysis of literature data (9, 10). While traditional review articles exist to summarize research progress, each review article focuses differently. Currently, there is still a lack of comprehensive, objective, and intuitive analysis of the evolution and trends of AI applications in AID. Bibliometric analysis based on quantitative analysis of literature can objectively and comprehensively describe the historical characteristics and development trends of this field (11).

**Figure 1:**
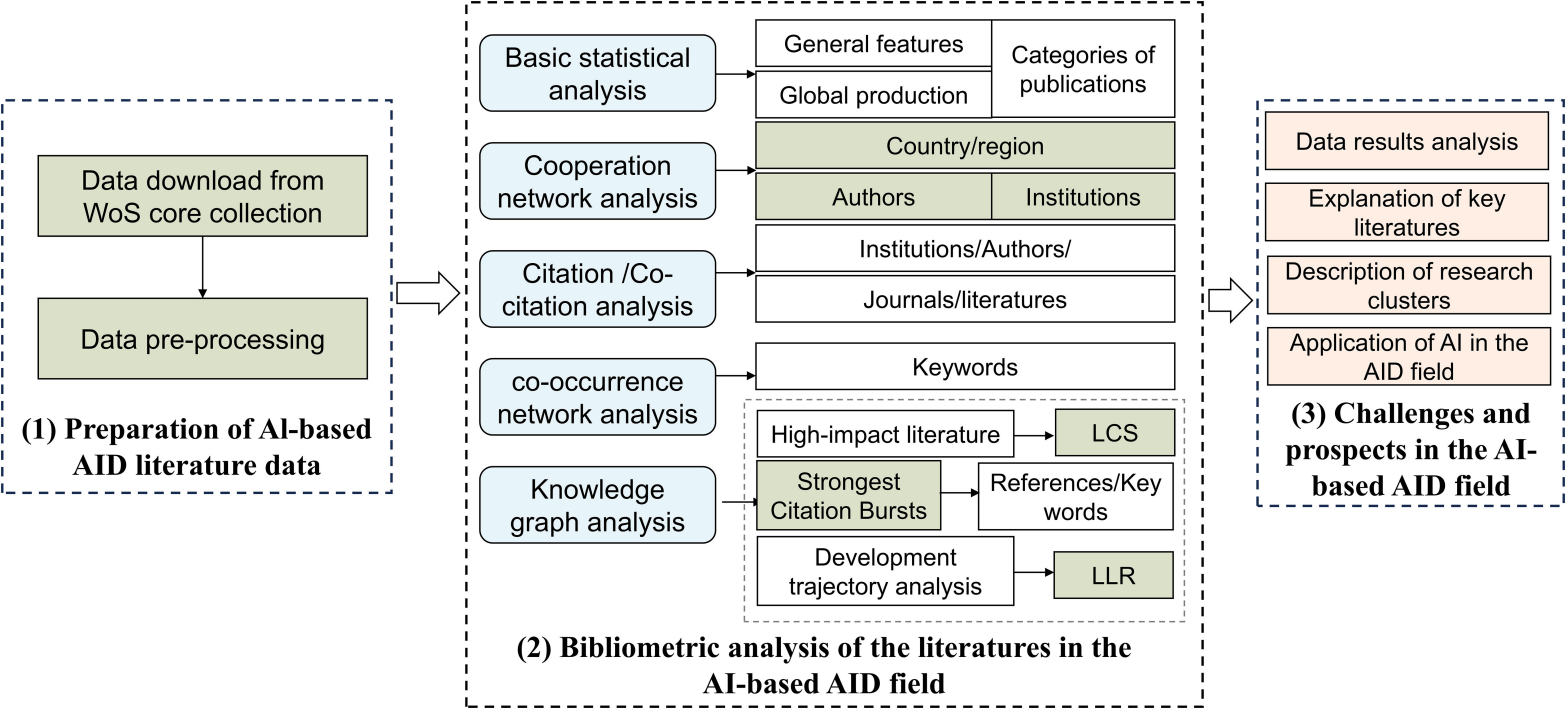
Analysis framework of this paper.

In this study, based on bibliometric analysis tools, we provided the research contents as follows (**Figure 1**): (1) comprehensively summarized the global development status in that field; (2) identified the high-productivity institutions, interested journals, highly cited authors, and pivotal milestone articles in that field; (3) visualized the evolution trajectory of research clusters in that field in a timeline format; (4) explored the current state of AI applications in the AID; (5) detailed the challenges and prospects.

## 2 Methods

### 2.1 Data collection

The Web of Science Core Collection (WoSCC) is a highly regarded database in the field of bibliometrics (11, 12), having gained significant recognition among scholars and serving as the primary data source informing this study. The search term strategy in this study was formulated by integrating reviews, bibliometric studies related to artificial intelligence and autoimmune diseases, as Medical Subject Headings (MeSH) terms in PubMed (5, 13). **Supplementary Table S1** provides a list of the search terms employed in this study.

The search was conducted on July 1, 2024, with the search terms limited to English language articles and reviews published between 2003 and the search date. After the retrieval and screening process, a total of 1,695 publications were identified, comprising 1,409 research articles and 286 reviews. The WoSCC was used to systematically collect data on publication countries/regions, institutions, journals, authors, articles, and keywords. Excel software (version 2021) was used to conduct summary and analysis.

### 2.2 Bibliometric analysis

CiteSpace is a Java application created by the research team led by Chen Chaomei at Drexel University (14). This software offers visualization techniques for publication data and has been widely acknowledged in the field of bibliometrics for providing objective analyses of academic frontiers (15, 16). In the CiteSpace analysis, co-occurrence networks were used to distinguish merged networks via color-coded nodes and edges. Burst detection, based on Kleinberg’s algorithm, was employed as an indicator of active topics (17). In this study, CiteSpace charted the literature network within the relevant field, offering analyses on research hotspots, frontier trends, and the evolution of knowledge structures.

VOSviewer, developed by Leiden University in the Netherlands, was utilized to extract and analyze elements within literature data such as authors, keywords, and institutions (18–20). It calculated the strength of their associations and presented them in a visual format. This process involved data extraction and preprocessing, association calculation, and visualization mapping to facilitate co-occurrence analysis, citation analysis, and clustering analysis. VOSviewer was utilized to create a node network, and relevant information was obtained through parameters such as link strength, cluster color, and node size analysis.

HistCite Pro 2.1 was employed to manage and analyze a large volume of literature data, excelling particularly in citation analysis (21). By utilizing HistCite, citation relationships between documents could be traced, elucidating the knowledge dissemination pathways and developmental trends within the research domain, thereby identifying highly significant literature in the field. In HistCite Pro 2.1, the “Limit” was set to 30, with the remaining settings kept at their default values. Subsequently, the “Make graph” option was selected to effectively and visually represent the interconnectedness within the research field, facilitating the efficient identification of key literature.

## 3 Results

### 3.1 General features of publications

Bibliometric analysis was able to quantitatively portray the developmental status within a specific academic research field. Our study revealed that over the past two decades, the total number of publications in this research field amounted to 1,695. Among these, 1,409 were research articles and 286 were reviews. These publications include the contributions of 10,915 authors from 7,070 institutions across 703 journals (**Supplementary Table S2**).

**Figure 2:**
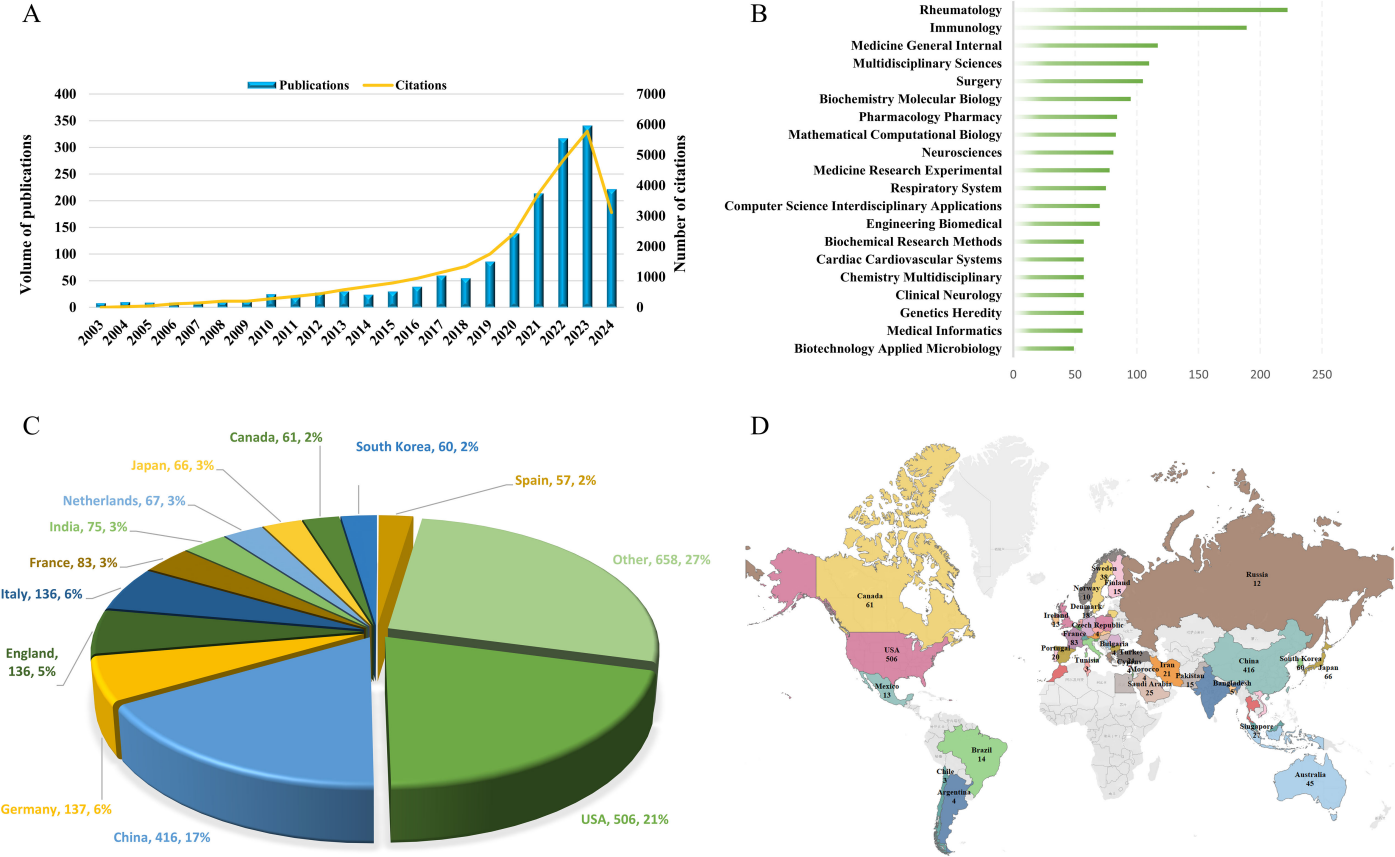
The evolution and distribution characteristics of publications. **(A)** The annual number of publications and citations over the past 20 years. **(B)** Top 20 Web of Science Categories for Publications. **(C)** Analysis of the publication numbers by country/region. **(D)** Global Distribution Overview of Publications.

As depicted in **Figure 2A**, we observed a rising trend in the number of publications over the past two decades, with particularly accelerated growth in the last five years. The count surpassed 100 for the first time in 2020, reaching 139 publications. By 2023, it had reached 341 publications, and in the first half of 2024 alone, the count had already reached 222 publications. In terms of citations, there was a noticeable upward trend in the number of citations in 2017, surpassing 1,000 and reaching 1,152. It is noteworthy that by 2023, the number of citations had reached 5,777.

### 3.2 Categories of publications

A thorough examination revealed that over the past two decades, publications on AI in AID spanned 124 Web of Science Categories. As illustrated in **Figure 2B**, the top 20 Categories with the highest publication counts are showcased. Among these, there are 5 Categories with over 100 publications, with *Rheumatology* leading at 222 publications, followed by *Immunology* with 189 publications. Subsequently, there were 117 publications in *Medicine General Internal*, 110 publications in *Multidisciplinary Sciences*, and 105 publications in *Surgery*.

### 3.3 Publication analysis of countries/regions

In the field of research, a total of 57 countries/regions contributed publications, with the USA having the highest number of publications at 506, accounting for 21% of the total, followed by China with 416 publications, making up 17%. Additionally, Germany had 137 publications, while England and Italy each had 136 publications. The pie chart in **Figure 2C** illustrates the distribution of publications by different countries/regions, revealing that the top 12 countries accounted for 73% of the total publications. The global distribution of publications by countries/regions, as depicted in **Figure 2D**, overall indicates that the United States and China made the most significant contributions to the field in terms of publication output. Unfortunately, it is noted that countries in Central Asia and regions in Africa have not yet ventured into this research field.

**Figure 3:**
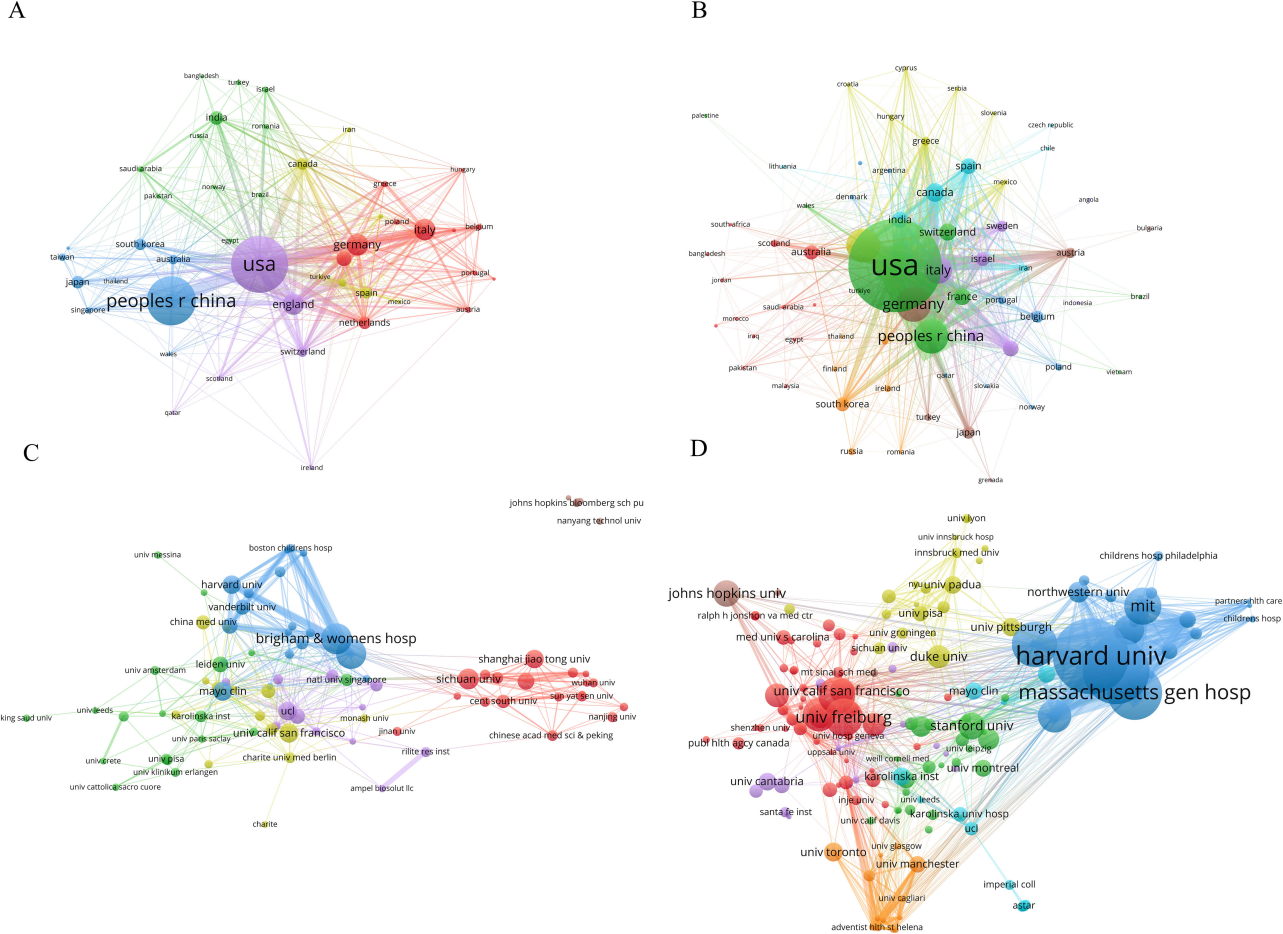
The co-authorship network among countries/regions and academic institutions. **(A)** Co-authorship Network of Countries/Regions. **(B)** Publication Citation Network of Countries/Regions. **(C)** Co-authorship Network of academic institutions. **(D)** Publication Citation Network of academic institutions. Network nodes symbolize publication or citation values, with larger nodes signifying higher values; Links between nodes reflect collaboration or citation strength, where thicker and darker lines denote stronger connections; Nodes of the same color are part of the same cluster, indicating similar characteristic.

Through the analysis of co-authorship relationships among countries/regions using VOSviewer, it was found that the USA had the highest total link strength, reaching 437, indicating a higher frequency and wider scope of collaboration with other countries (**Figure 3A**). In terms of publication citations, a minimum citation threshold of no less than 5 times was set, and a country/region citation network map was generated (**Figure 3B**). In this network, node size represents citation counts, with the top five countries in citation counts being the USA, Germany, China, England, and Italy, with citation frequencies of 13,515, 3,425, 3,298, 3,277, and 2,436 respectively. Furthermore, through the analysis of total link strength, the USA, Italy, and China ranked in the top three with values of 1,849, 1,040, and 1,023 respectively, indicating the wider dissemination of publication citations from these three countries/regions.

### 3.4 Publication analysis of academic institutions

Various institutions leverage their expertise in clinical medicine, data algorithms, and biological research to collaboratively consolidate resources and knowledge. Setting the publication threshold at a minimum of 8 articles, a collaboration network comprising 88 academic institutions was established (**Figure 3C**). Node size represented publication output, with Brigham & Women’s Hospital, Harvard Medical School, Sichuan University, Mayo Clinic, and University of California San Francisco ranking as the top five institutions, with publication counts of 37, 36, 25, 23, and 23 respectively. Moreover, in this network, Brigham & Women’s Hospital, Harvard Medical School, and Massachusetts General Hospital ranked among the top three for total link strength, with values of 91, 67, and 60 consecutively.

By focusing on citations, with a minimum threshold of 100 citations, a total of 223 academic institutions were identified in the network graph (**Figure 3D**). The analysis revealed that Harvard University claimed the top position with 1,567 citations, followed by Brigham & Women’s Hospital with 1,372 citations and Massachusetts General Hospital with 1,129 citations. In the citation network graph, Brigham & Women’s Hospital was identified as the institution with the highest total link strength, with a value of 743. Massachusetts General Hospital and Harvard University recorded 638 and 597, respectively. These findings indicate that these three institutions occupy a dominant position within the field.

### 3.5 Publication analysis of authors

**Figure 4:**
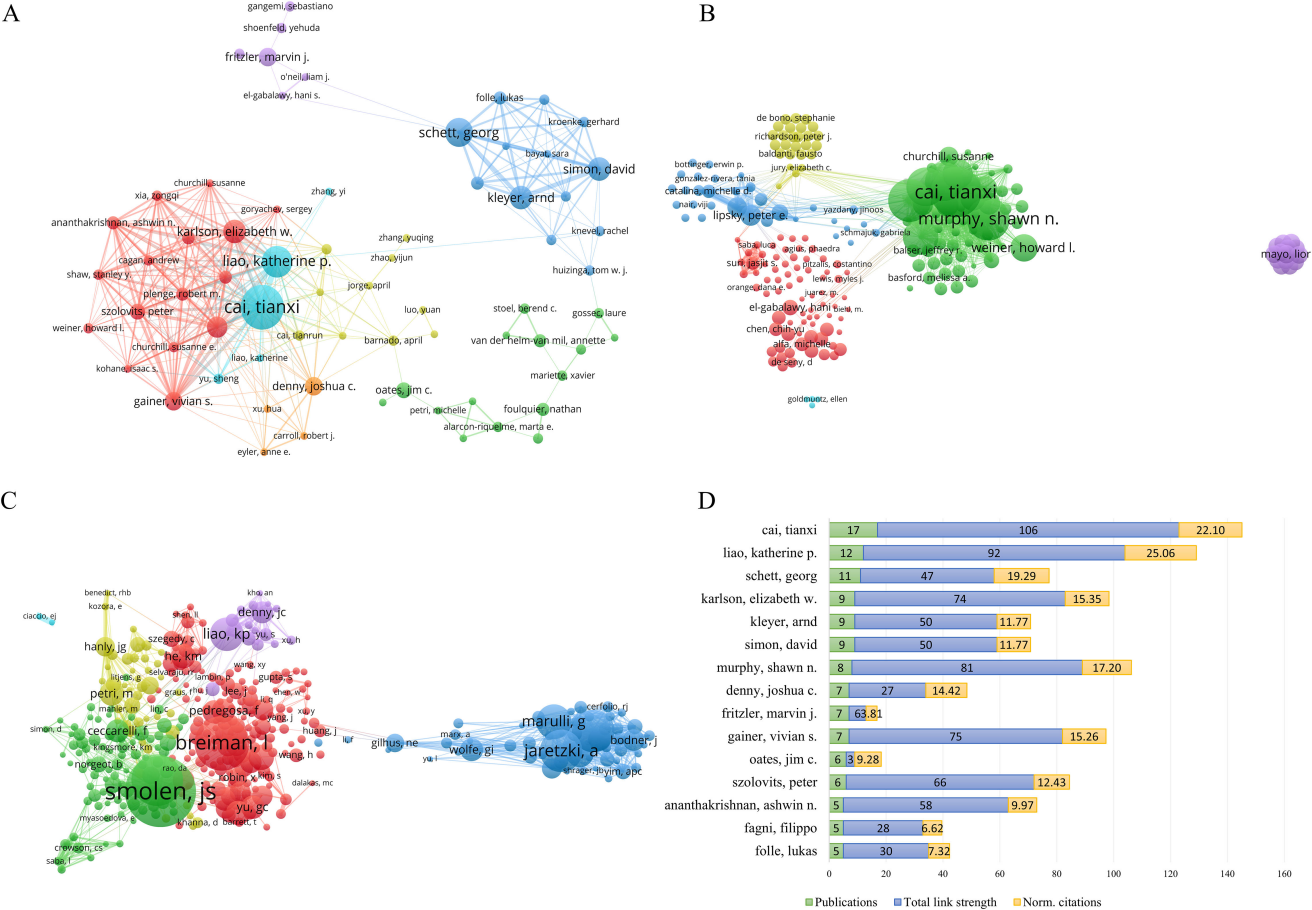
Author analysis of publications. **(A)** Network of co-authors of publications. **(B)** Citation network of authors in the field. **(C)** Co-citation network of authors. **(D)** Analysis of Total link strength and Normalized citations among the top 15 authors based on publication count.

Interdisciplinary collaboration among authors overcomes professional barriers and drives progress in the field. Based on a minimum threshold of 3 publications, an analysis was performed to construct a co-authorship network graph for 323 authors (**Figure 4A**). The analysis revealed the existence of distinct co-author clusters, with notable prominence observed in clusters spearheaded by Gainer, Vivian S. and Karlson, Elizabeth W., Cai, Tianxi and Liao, Katherine P., as well as Kleyer, Arnd, Simon, David, and Schett, Georg. These findings suggest that the aforementioned individuals demonstrated higher levels of involvement and collaboration in research activities within their respective clusters. With regard to the presentation of citations, **Figure 4B** depicts the citation network graph, wherein the nodes represent the citation counts. Cai, Tianxi was the most highly cited author, with 955 citations, and also ranked first in total link strength within the network, with a value of 106. A co-citation network graph was constructed based on a minimum citation frequency threshold of 20 times, comprising a total of 219 authors (**Figure 4C**). In this co-citation network, the highest number of citations were attributed to Smolen, JS; Breiman, L.; and Aletaha, D., with respective citation counts of 177, 136, and 129.

We also analyzed the total link strength and *norm. Citations* of the top 15 authors in terms of publication volume (**Figure 4D**). Our results revealed that the author with the highest publication volume was Cai, Tianxi, with 17 articles, and the highest total link strength of 106. However, Cai, Tianxi ranked second in the *norm. Citations* with 22.10. The author ranked first in the norm. The citation was Liao, Katherine P., with a publication volume of 12 and a total link strength of 92, which placed them second. This indicates the significant influence of Cai, Tianxi, and Liao, Katherine P. in the field of study.

### 3.6 Publication analysis of journals

**Figure 5:**
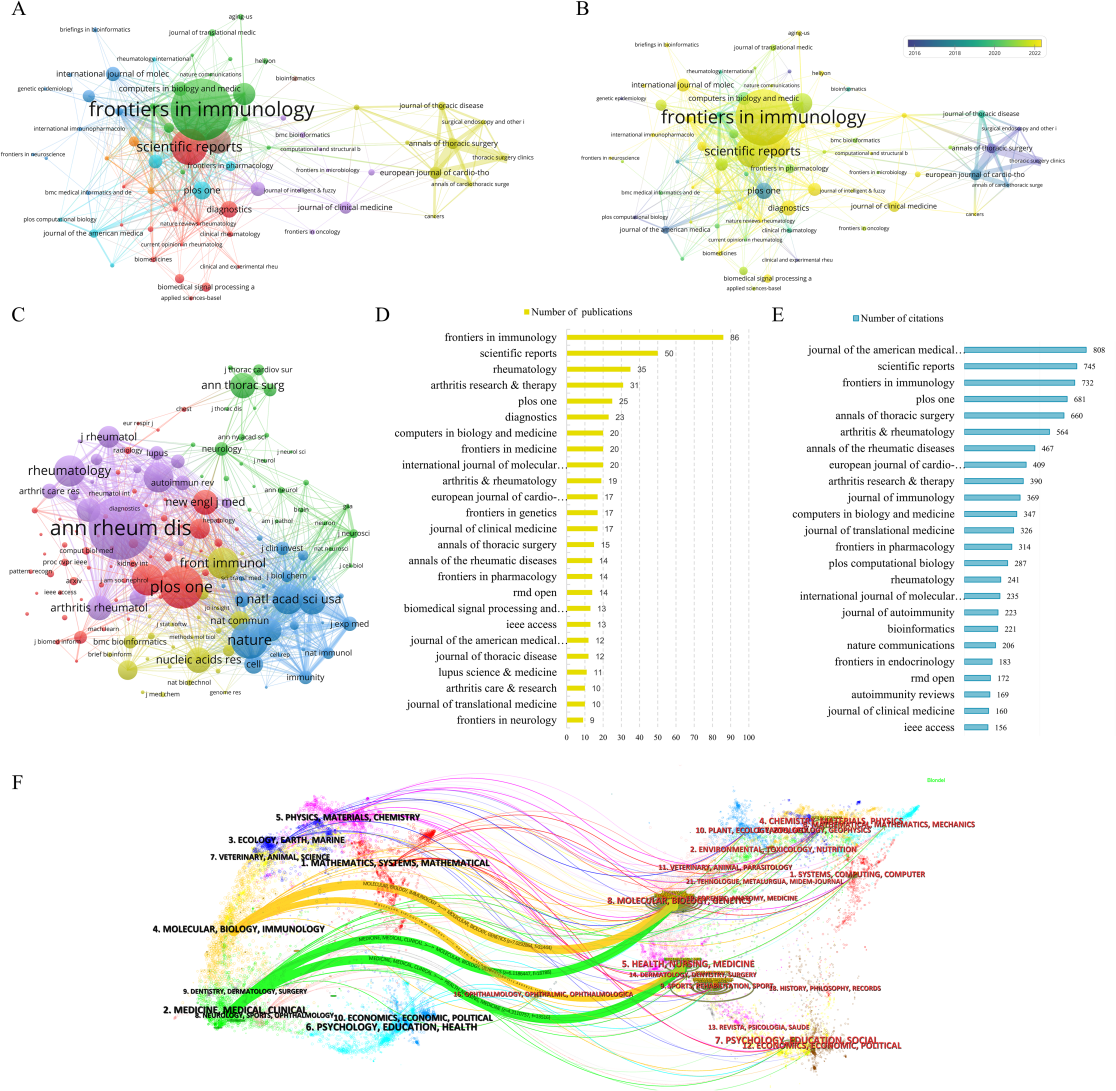
Journals analysis of publications in research fields. **(A)** Citation network analysis of journals. **(B)** Analysis of Journal Citation Years in the Field. **(C)** Co-citation network analysis of journals. **(D)** The 25 Journals with the Highest Number of Publications. **(E)** Top 25 journals in terms of citations. **(F)** The dual-map overlay of journals.

Through an analysis utilizing VOSviewer of journal citation networks, it was observed that *Autoimmunity Reviews*, *Annals of Thoracic Surgery*, *European Journal of Cardio-Thoracic Surgery*, *Scientific Reports*, and *Lupus Science & Medicine* emerged as the top five journals based on total link strength, achieving scores of 134, 114, 111, 105, and 99 respectively (**Figure 5A**). These findings suggest a pronounced citation relationship with other journals. Moreover, an examination of the temporal aspect of journal citations, as illustrated in **Figure 5B**, revealed that yellow nodes denote journals that have displayed heightened activity in the field in recent years, whereas progressively bluer nodes signify earlier periods of relative activity for these journals. Furthermore, by setting the minimum citation threshold to no fewer than 100, a total of 154 core journals were utilized to construct the co-citation network (**Figure 5C**). The results indicated that the total link strengths for *Annals of the Rheumatic Diseases*, *Nature*, *Proceedings of the National Academy of Sciences of the United States of America*, *PLOS One*, and *Journal of Immunology* were 88,303, 72,645, 60,534, 58,811, and 57,919, respectively.

In the realm of academic publication output, *Frontiers in Immunology* emerged as the frontrunner with 86 articles, closely trailed by *Scientific Reports* with 50 articles, *Rheumatology* with 35 articles, *Arthritis Research & Therapy* with 31 articles, and *PLOS One* with 25 articles. Additionally, in terms of scholarly impact measured by citations, the *Journal of the American Medical Informatics Association* claimed the top position with 808 citations, followed by *Scientific Reports* with 745 citations, *Frontiers in Immunology* with 732 citations, *PLOS One* with 681 citations, and *Annals of Thoracic Surgery* with 660 citations (**Figures 5D, E**).

The dual-map overlay function illustrated the distribution of citing and cited journals, revealing the interdisciplinary connections between the research field and other disciplines. In **Figure 5F**, the dual-map overlay showed citing journals on the left side and cited journals on the right side. Two significant citation pathways were observed. The yellow path indicated that journals in the fields of molecular biology and immunology tended to cite publications from molecular biology, genetics, as well as health, nursing, and medical disciplines. The green path, on the other hand, suggested that medicine, medical, and clinical journals were inclined to cite publications from molecular, biology, genetics, as well as health, nursing, and medical fields.

### 3.7 Historical trajectory of the research field

#### 3.7.1 Analysis of influential literature

Using HisCite, a citation history map of research articles was generated. **Supplementary Table S3** listed literature of significant reference value based on the Local Citation Score (LCS) and Global Citation Score (GCS). The LCS indicates the frequency of citations a work receives within a specific research database or field, showcasing its influence within that scope. The study by Rea F et al. in 2006 reported the experience of thymectomy in myasthenia gravis patients using the “da Vinci” robotic system, which received the highest LCS of 45 (22). This was followed by the comparative study of robotic versus non-robotic thoracoscopic thymectomy by Rückert JC et al. in 2010, and the research by Yuanfang Guan et al. in 2019 on predicting the response to anti-tumor necrosis factor drugs in rheumatoid arthritis patients by integrating clinical and genetic markers using machine learning, with LCS values of 38 and 34, respectively (23, 24).

**Figure 6:**
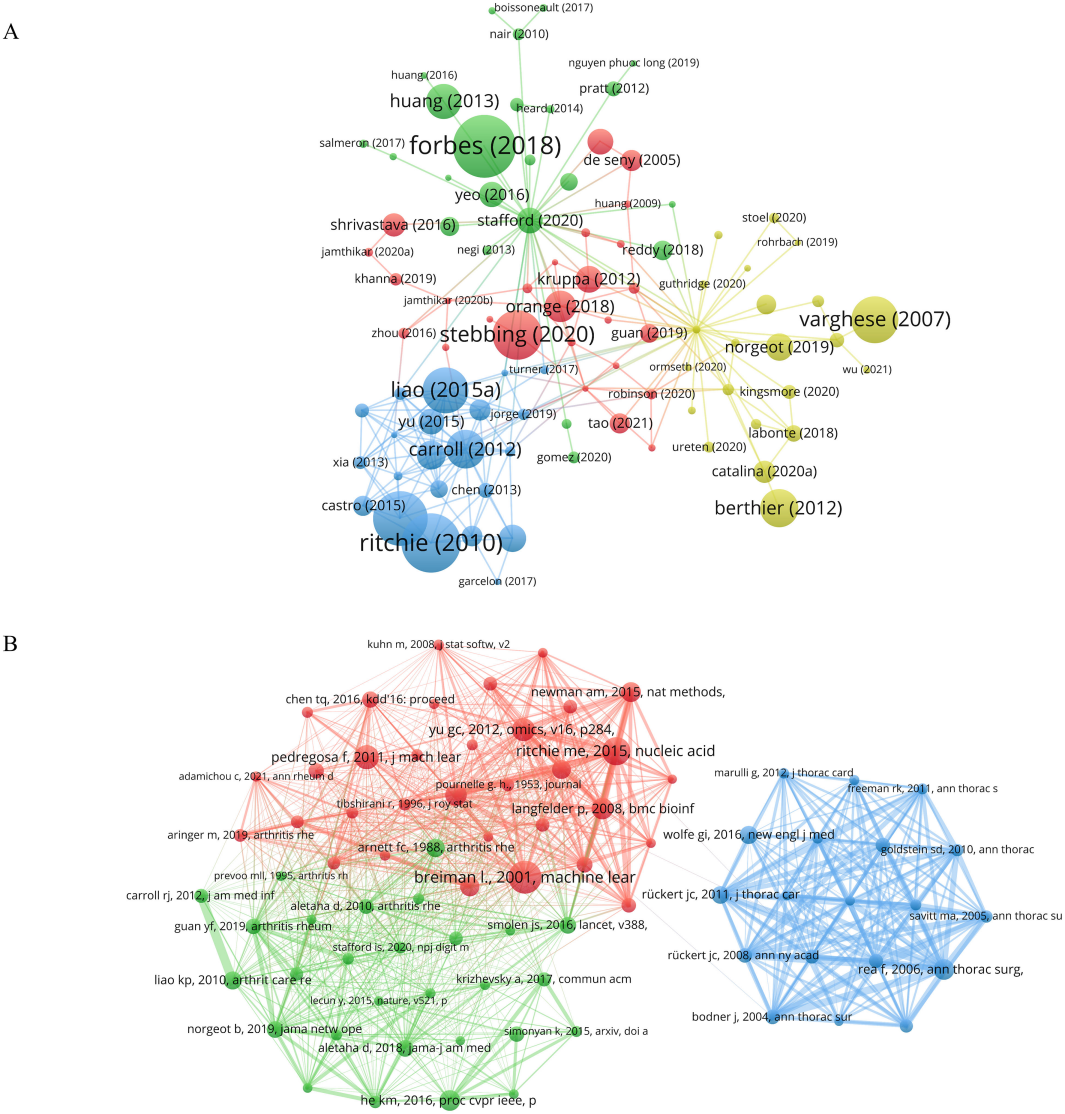
Analysis of publications in the field of research. **(A)** Network diagram of literatures citation analysis. **(B)** Network diagram for co-citation analysis of literatures. Node size indicates the citation count of the literature. The same color represents the same cluster.

In VOSviewer, with the citation threshold set to no fewer than 25 times, a citation network graph comprising 309 publications was constructed (**Figure 6A**). The nodes in the graph represent the citation counts, with Forbes (2018) (25), Ritchie (2010) (26), and Ford (2016) (27) ranking top three with 254, 240, and 220 citations, respectively. Additionally, we analyzed the co-citation of literature in the research field by setting the citation frequency threshold to no fewer than 20 times, resulting in the selection of 80 publications for creating a co-citation network (**Figure 6B**). Through the analysis of the total link strength, it was revealed that the publications by Rea F (2006) (22), Ritchie ME (2015) (28), and Rückert JC (2011) (24) held prominent positions in the network, with total link strengths of 270, 235, and 204, respectively.

#### 3.7.2 Analysis of literature development characteristics

**Figure 7:**
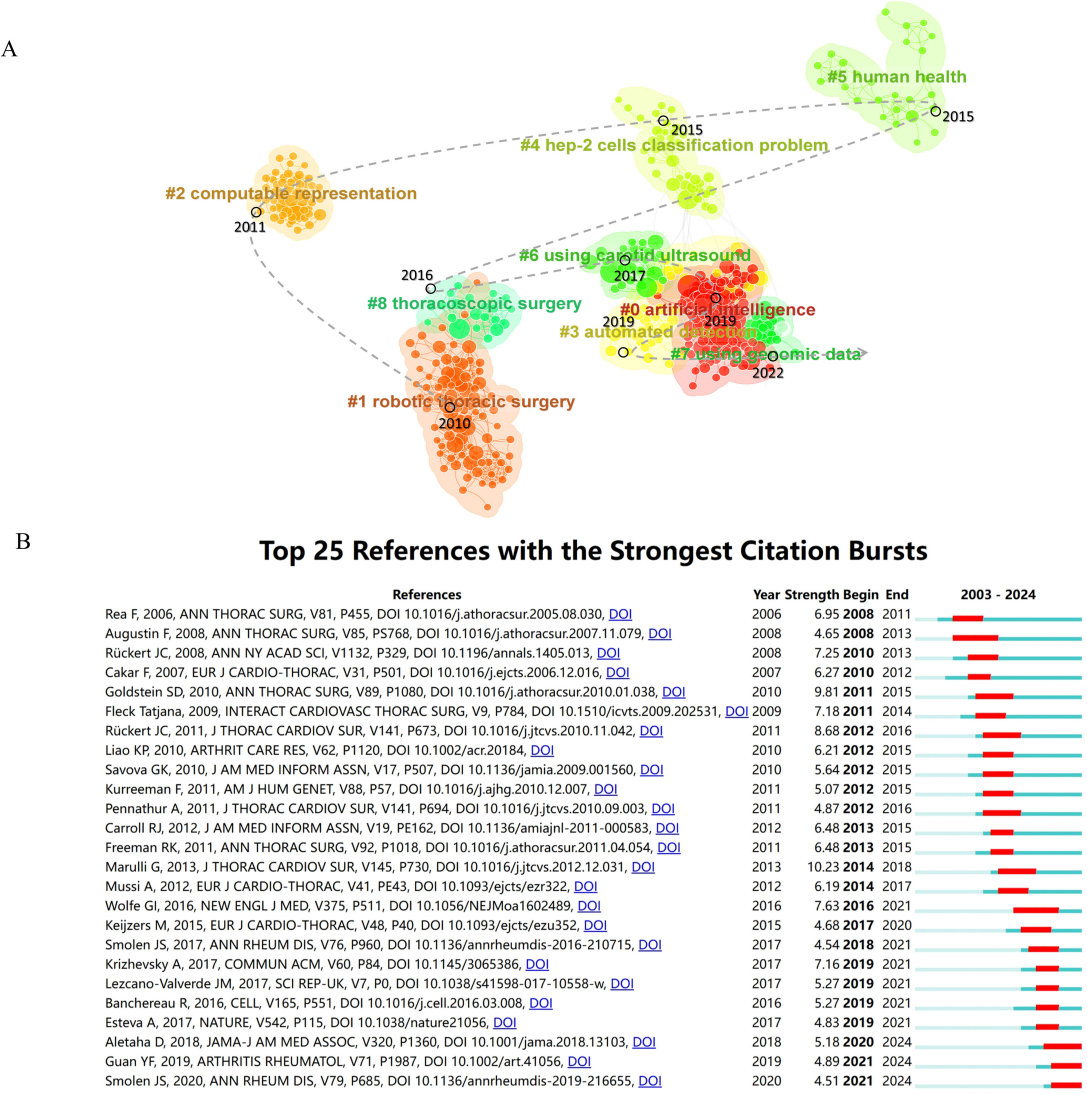
Analysis of the development characteristics of literature clusters. **(A)** Literature clustering analysis and its evolutionary trajectory. **(B)** Top 25 References with the Strongest Citation Bursts.

In bibliometrics, Citespace was used to cluster all references using the log-likelihood ratio (LLR) algorithm, resulting in the visualization of the top 9 clusters in **Figure 7A**. By plotting the average cluster years, we mapped the developmental trajectory of clusters in this research field. Furthermore, based on Citespace’s clustering criteria, silhouette values close to 1 indicated high cohesion and separation within the clusters, while values above 0.7 signified convincing clustering. **Supplementary Table S4** detailed the cluster names chronologically.

In 2010, the establishment of Cluster #1, Robotics Thoracic Surgery, marked a significant milestone in the field, with a primary focus on the utilization of robotic thymectomy in patients with myasthenia gravis (24, 29, 30). The subsequent year, 2011, witnessed the emergence of Cluster #2, Computable Representation, which delved into the utilization of electronic health records and algorithms to elevate the precision in the identification of conditions such as rheumatoid arthritis (31, 32). Fast forward to 2015, Clusters #4 and #5 came into existence. The former, Cluster #4, highlighted the pivotal role of HEp-2 cell classification in the realm of autoimmune disease diagnosis (33), whereas Cluster #5, Human Health, provided insights into the intricate relationship between the immune epitope database (IEDB) and gut microbiota. This cluster shed light on the dynamic interplay between gut microbiota and the host immune system, contributing to a deeper comprehension of immune system functionality (34–36). In 2016, Cluster #8 (Thoracoscopic Surgery) was established, focusing on conducting thymectomy using a combination of robotic and video-assisted thoracoscopic surgery (VATS) techniques (37). Cluster #6 (Using Carotid Ultrasound) emerged in 2017, with a focus on reporting the cardiovascular risk assessment in autoimmune disease patients through the use of carotid ultrasound B-mode imaging (38). Cluster #0 (Artificial Intelligence) and Cluster #3 (Automated Detection) shared many similar research foundations and were simultaneously formed in 2019. Cluster #0 focused on machine learning, aiming to better understand the complex mechanisms of autoimmune diseases by organizing and analyzing large volumes of clinical and immunological data to enhance diagnostic and predictive capabilities (23, 39, 40). Additionally, Cluster #3 centered on the identification of biomarkers associated with autoimmune diseases to gain further insights into the mechanisms of disease onset (39, 41). The final cluster to emerge in 2022, Cluster #7 (Using Genomic Data), originated from the machine learning cluster. The primary objective of this cluster was to employ machine learning techniques for the analysis of both traditional clinical data and novel genomic data, with the aim of developing predictive models that could enhance the clinical diagnosis and treatment of autoimmune diseases such as lupus nephritis and systemic lupus erythematosus (39, 42–44).

#### 3.7.3 References with the strongest citation bursts

CiteSpace utilized an algorithm to identify the top 25 references with the strongest citation bursts, setting a threshold for a minimum burst duration of 3 years (**Figure 7B**). The case report by Rea F (2006) on thymectomy using the “da Vinci” robot for treating myasthenia gravis patients marked the earliest citation burst, lasting for 3 years (22). In 2013, Marulli G reported on the surgical and neurologic outcomes after robotic thymectomy in 100 consecutive patients with myasthenia gravis, which achieved the highest burst strength in the field at 10.23 and lasted for 4 years (30). Additionally, ongoing momentum was observed in some references, including recommendations by Aletaha D (2018) (45) and Smolen JS (2020) (46) on the diagnosis and management of rheumatoid arthritis, including personalized therapy through predictive markers, and Guan YF (2019) utilizing machine learning to predict drug responses in rheumatoid arthritis patients (23).

### 3.8 Keyword-based topic evolution and frontiers

#### 3.8.1 Co-occurrence network analysis of keywords

**Figure 8:**
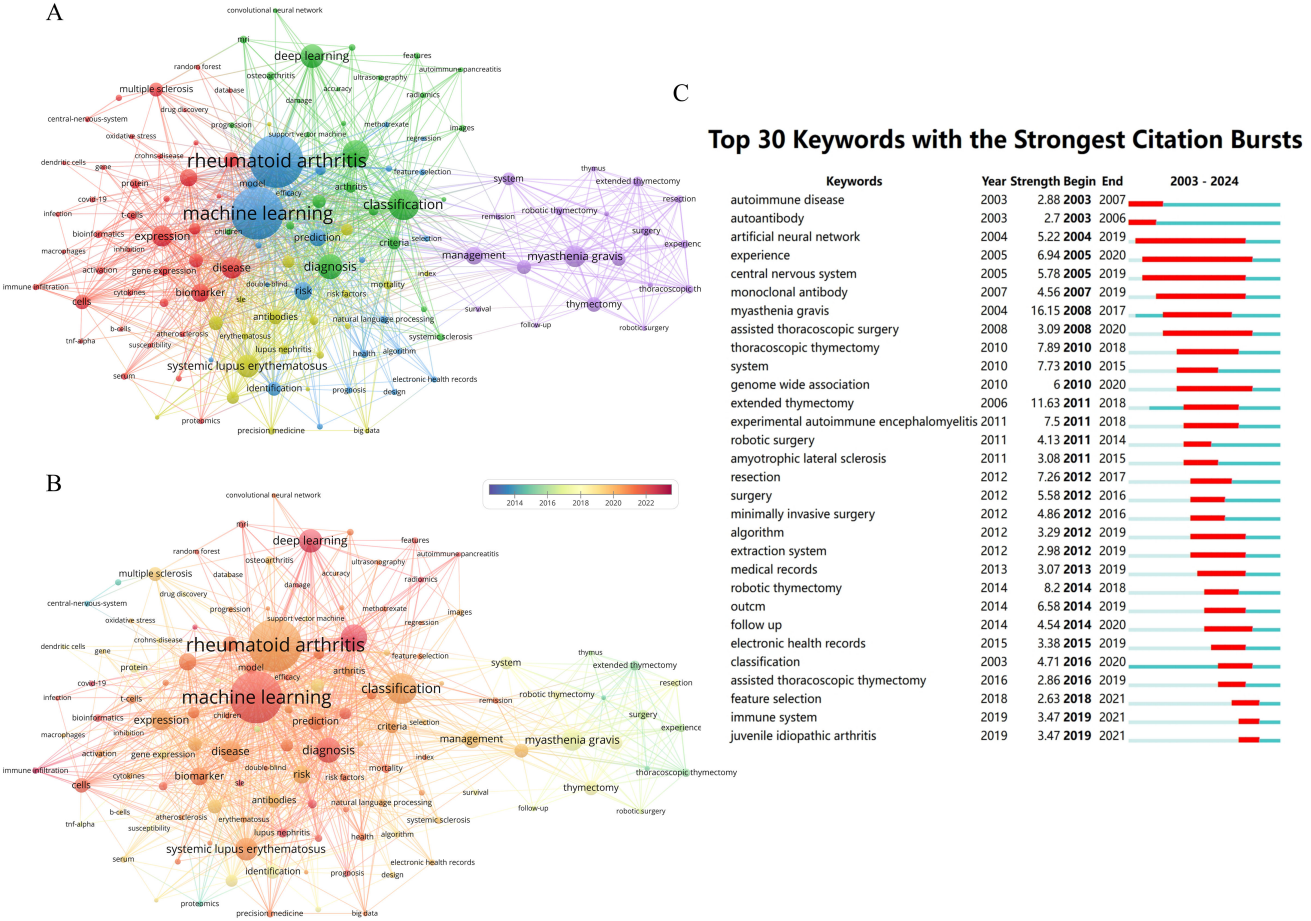
Keywords network analysis in the field. **(A)** Keyword co-occurrence network analysis. **(B)** Analysis of Keywords co-occurrence networks in a temporal perspective. **(C)** Top 30 Keywords with the Strongest Citation Bursts.

The co-occurrence analysis reveals the hotspots and overall correlations in the research field. Utilizing VOSviewer, a co-occurrence network consisting of 130 keywords is constructed with a minimum co-occurrence frequency of not less than 15 words (**Figure 8A**). Upon analysis, it is observed that the most frequent co-occurring keywords in the network are “machine learning” with 372 occurrences and “rheumatoid arthritis” with 208 occurrences. Furthermore, the network exhibits distinct clustering, with a total of 5 clusters identified. Among these, 4 clusters demonstrate extensive interconnections, while the cluster represented by purple nodes, centered around “myasthenia gravis,” shows limited intersection with other clusters. This cluster primarily highlights the application of AI-based robotics in thymectomy procedures.

Due to different time periods, the academic frontiers focus on different hot topics. By analyzing the annual changes of the co-occurrence network, we found that the cluster represented by “thymectomy” was generally active before 2018, as shown in **Figure 8B**. Additionally, we have also observed that “machine learning” and “deep learning” are becoming hot topics in this research field, particularly in the study of key diseases such as rheumatoid arthritis, systemic lupus erythematosus, lupus nephritis, multiple sclerosis, among others.

#### 3.8.2 Strongest citation bursts analysis of keywords

Keyword bursts signify a significant increase in the frequency of specific terms during a defined time period. Analyzing these bursts aids researchers in identifying emerging trends, predicting future directions, and guiding research decisions. Applying a threshold of no less than 3 years, Citespace conducted an analysis of the Strongest citation bursts of keywords based on Kleinberg’s burst detection algorithm (47). In **Figure 8C**, the top 30 keywords were listed, with “autoimmune disease” and “autoantibody” displaying the earliest onset of Strongest citation bursts, dating back to 2003. The keyword showing the highest burst strength was “myasthenia gravis” at 16.15. Furthermore, the keywords “artificial neural network” and “experience” maintained the longest durations, lasting for 15 years with burst intensity values of 5.22 and 6.94, respectively. Over the past five years, “immune system” and “juvenile idiopathic arthritis” have also emerged as focal points of research interest in the field.

#### 3.8.3 Dynamic process of research topics

**Figure 9:**
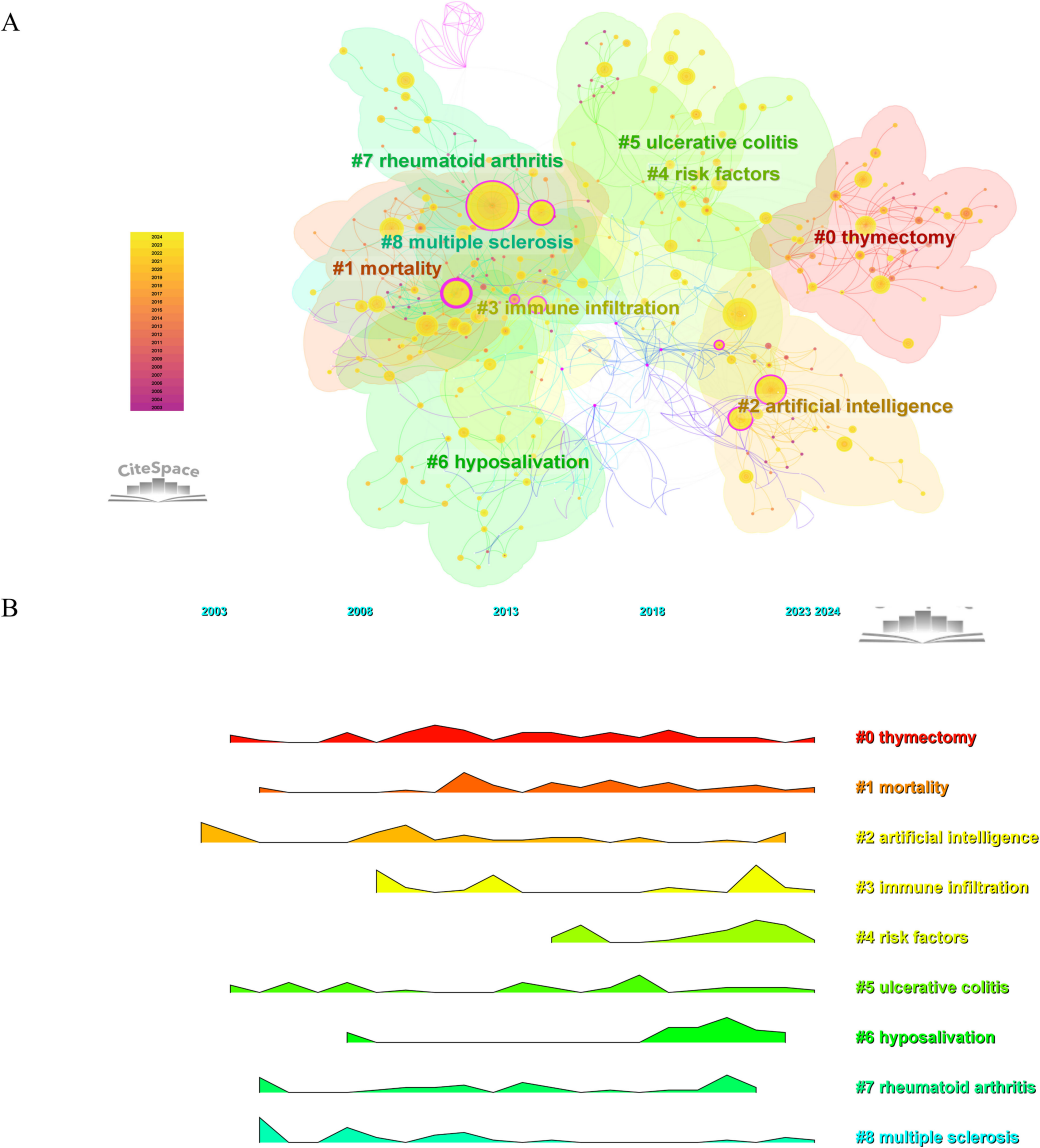
Keywords clustering analysis and development trajectory. **(A)** Keyword-based cluster analysis. **(B)** Landscape map analysis of keyword clusters.

Clustering was performed using the LLR algorithm on a keyword co-occurrence network. The top 9 clusters depicted in **Figure 9A** include: #0 thymectomy, #1 mortality, #2 artificial intelligence, #3 immune infiltration, #4 risk factors, #5 ulcerative colitis, #6 hyposalivation, #7 rheumatoid arthritis, and #8 multiple sclerosis. The central item in Cluster #8 is (2005) with a centrality of 0.26, followed by (2005) in Cluster #7 with a centrality of 0.20, and (2005) in Cluster #1 with a centrality of 0.17. **Figure 9B** displayed the cluster landscape based on keyword co-occurrence, allowing for the observation of the distribution changes of each cluster from 2003 to 2024.

## 4 Discussion

### 4.1 Global development issues in the research field of AI-AID

In this study, we observe an increasing trend in the number of publications in the field over the past twenty years, with a particularly rapid growth in the past five years. The number of publications surpassed 100 for the first time in 2020, reaching 139, and by 2023 had escalated to 341. In just the first half of 2024, the number had reached 222. This growth trend indicates a continuous rise in research interest in the field, possibly influenced by factors including advancements in related technologies, increased societal attention, and heightened research investments. However, we must also acknowledge that this rapid growth trend may bring about certain challenges. With the increasing number of publications, there could be variations in research quality, necessitating more rigorous peer review mechanisms to ensure the reliability and effectiveness of research.

Both the United States and China have made substantial contributions in this field, however, there is an evident developmental imbalance among countries and regions worldwide. Patients with AID in these two countries benefit from more accurate diagnosis and effective treatment, ultimately improving their quality of life and reducing the suffering and societal burden imposed by these illnesses. Publications and collaborations in the field from African and Central Asian countries are relatively scarce, potentially due to limited healthcare resources and research funding in these regions, leading to disparities in the application of artificial intelligence in medicine. Further analysis supports the notion that countries with fewer publications or citations are predominantly from middle- to low-income or low-income areas.

Although automated, AI-driven medical applications can overcome barriers to healthcare equity in middle- to low-income countries, AI medical models trained in high-income regions may exhibit suboptimal performance when applied in middle- to low-income countries due to the phenomenon of “data set shift,” where differences in patient populations, clinical practices, and healthcare systems lead to discrepancies (48, 49). Hence, it is essential for the future to enhance collaboration with middle- to low-income countries to ensure model generalizability by collecting data from diverse and representative populations across various countries and regions, thereby preventing data set shifting (50).

### 4.2 Current status of AI in autoimmune diseases

**Figure 10:**
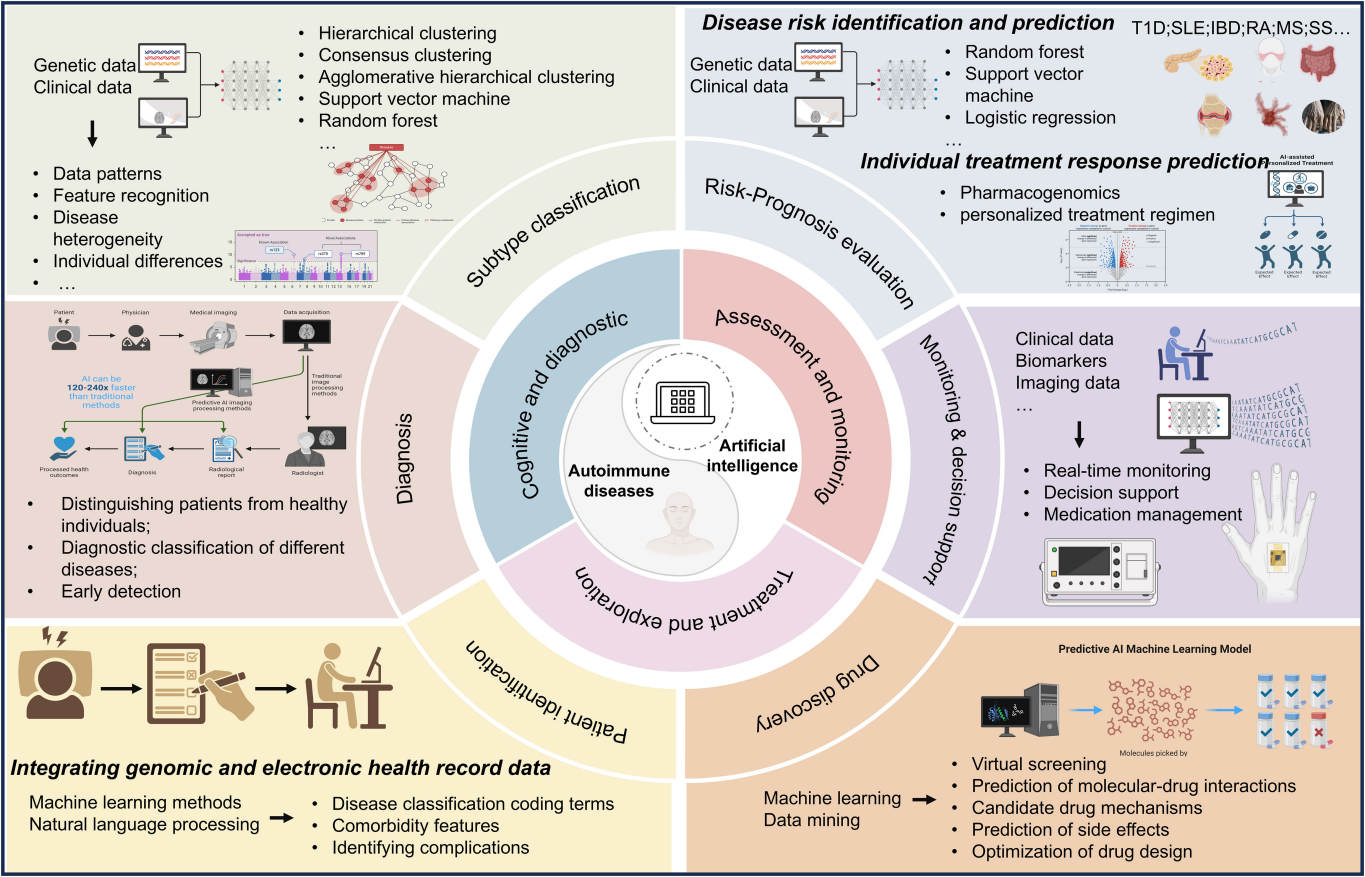
The scheme of major applications of artificial intelligence technology in autoimmune diseases.

Through the analysis of key terms in the research field, the main applications of AI in autoimmune diseases have been identified. AI is most commonly utilized in diseases such as multiple sclerosis, rheumatoid arthritis, and systemic lupus erythematosus, possibly due to the high prevalence and significant clinical attention these diseases receive. The application scenarios of AI in this field encompass six main areas: patient identification, risk factors and prognosis assessment, diagnosis, classification of disease subtypes, monitoring and decision support, and drug discovery (**Figure 10**) (5, 51, 52).

#### 4.2.1 Patient identification

Electronic medical records (EMRs) have the capability to integrate complex medical histories, facilitating the recording, supervision, and extraction of clinical data (53–59). Meanwhile, efforts are being made in some studies to integrate genomic and EMR data, advancing the field of precision medicine (60, 61). Machine learning methodologies are utilized for the identification of individuals diagnosed with autoimmune disorders from electronic health records, with natural language processing playing a crucial role in the recognition of associated comorbidities such as celiac disease, osteoarthritis, and rheumatoid arthritis-related complications (musculoskeletal symptoms, infections) (62–66). Furthermore, it is possible to reveal the comorbidity characteristics of autoimmune diseases with other factors, including the co-occurrence of hypertension and autoimmune disorders heightening the risk of Alzheimer’s disease, as well as polycystic ovary syndrome (PCOS) impacting immune system disorders (67, 68). Additionally, enhancements in algorithm efficiency contribute to reducing the elevated error rates stemming from inconsistent terminologies in the International Classification of Diseases coding (69–71).

#### 4.2.2 Risk factors and prognosis assessment

The prediction of autoimmune disease risk and identification of new risk factors involve the utilization of genetic data, clinical data, and other resources (72–75). Common methods include random forests, support vector machines, and logistic regression (76, 77). Analysis of genetic data can reveal disease-associated genetic variations, elucidating the genetic basis of the disease and predicting individual disease risk. Integration and analysis of clinical data can uncover the relationships between clinical characteristics and autoimmune diseases. Feature selection algorithms and similar methods enable the identification of factors closely associated with disease risk from a vast amount of data. These approaches are applied in the prediction of diseases such as inflammatory bowel disease (IBD) (78, 79), type 1 diabetes (T1D) (80–82), rheumatoid arthritis (RA) (83–86), systemic lupus erythematosus (SLE) (87, 88), Sjögren’s syndrome (SS) (76)and multiple sclerosis (MS) (89–91).

In disease progression and prognosis, commonly used methods include support vector machines, random forests, and neural networks combined with clinical data. Research on disease progression and treatment encompasses conditions such as psoriasis, lupus nephritis, rheumatoid arthritis, inflammatory bowel disease, and celiac disease (92–98). Furthermore, machine learning can utilize patient treatment response data to predict the effectiveness of different treatment regimens for individual patients, such as the response to anti-rheumatic drugs, adalimumab, and etanercept in the treatment of rheumatoid arthritis (99, 100). It is worth mentioning that drug prognostic responses in systemic lupus erythematosus, multiple sclerosis, ankylosing spondylitis, inflammatory bowel disease, and psoriasis are gradually being applied (101–105). Genomics is also being increasingly integrated into this research area, exemplified by studies on drug genomic research related to methotrexate treatment response in patients with RA (106).

#### 4.2.3 Diagnosis

AI plays a crucial role in disease diagnosis, encompassing aspects such as distinguishing patients from healthy controls, diagnostic classification of different diseases, and early detection. Some studies focus on distinguishing specific diseases such as RA and SLE from healthy controls (107–110), while others aim to differentiate between diseases with similar symptoms, including myalgic encephalomyelitis and chronic fatigue syndrome, multiple sclerosis, celiac disease, irritable bowel syndrome, and psoriasis (111–113). Additionally, there are research efforts dedicated to the early diagnosis of delayed-onset diseases like MS and RA (114, 115).

#### 4.2.4 Classification of disease subtypes

In the classification of subtypes of autoimmune diseases, machine learning techniques are employed to utilize patient clinical data, genetic data, and other resources to identify potential subtypes by learning patterns and features in the data, thereby understanding disease heterogeneity and individual differences. Common methods include hierarchical clustering, consensus clustering, agglomerative hierarchical clustering, support vector machines, and random forests, among others. This research encompasses subtype classification of diseases such as RA, IBD, MS, SLE, SS, and idiopathic myositis (IM) (116–119).

#### 4.2.5 Monitoring and decision support

Artificial intelligence technology is utilized to analyze patients’ clinical data, biological markers, and imaging data, enabling real-time monitoring and tracking of autoimmune diseases. This assists healthcare professionals in promptly identifying the progression status and changing trends of diseases. Machine learning is applied in the prediction of blood glucose levels, identification of hypoglycemic events, and decision support in diseases such as T1D (120, 121). Digital remote monitoring interventions are employed for disease activity monitoring in patients with inflammatory arthritis (122). Furthermore, AI is utilized for MRI monitoring in MS, treatment compliance in IBD, and drug management (123–125). AI integrated diverse data sources to achieve precise diagnosis and risk prediction, assisted in developing personalized treatment plans, and improved treatment outcomes while utilizing smart devices for real-time patient monitoring, thus providing continuous health management.

#### 4.2.6 Drug discovery

In the field of drug discovery for autoimmune diseases, AI is employed to accelerate and enhance the drug development process using machine learning, data mining, and artificial intelligence technologies. This encompasses virtual screening, prediction of molecular-drug interactions, forecasting candidate drug mechanisms and side effects, as well as drug design optimization. For SLE, some biologics have been approved, and molecular analysis has identified specific molecular features and genetic markers of the disease (126–128). The treatment of RA includes various traditional medications and TNF antagonists, which, through genomic and transcriptomic analyses and machine learning models, guide the prediction of TNF treatment response and the development of new medications (129–132). Personalized drug discovery for other autoimmune diseases is also progressively advancing in relevance (3).

### 4.3 Challenges and prospects

AI shows potential in the field of autoimmune diseases, but it faces numerous problems and challenges. In terms of models, there is insufficient validation, necessitating enhanced cross-validation and independent testing to improve accuracy and reliability. Complex models are in demand, yet they encounter difficulties in computational power, methods, and data management. Data integration from multiple sources is challenging and requires standardization. Additionally, there is a lack of comprehensive “healthy” immune response models to differentiate between normal and diseased states. In treatment, the testing of combination therapies is hindered by numerous possibilities, requiring improved computational methods to assess their effectiveness and safety, while regulatory acceptance of digital evidence remains uncertain. Autoimmune diseases are complex and heterogeneous, posing difficulties in model construction and precise treatment implementation. Understanding disease mechanisms and developing advanced algorithms are crucial to address these challenges and drive progress in the field. Furthermore, there are significant privacy and security risks in medical health data, making the protection of patient data privacy a critical consideration when utilizing AI for autoimmune disease data processing (3, 5, 133–137). To address these challenges, future research should enhance cross-validation and independent testing, improve computational power, optimize data management and methods, promote data standardization, and develop universal and practical models. Furthermore, researchers must prioritize data privacy protection to ensure legal and compliant usage. Interdisciplinary collaboration should follow ethical guidelines to safeguard patients from potential harm and support the safe and reliable advancement of the field.

AI shows significant potential in the field of autoimmune diseases. The upcoming directions encompass enhanced diagnostic precision and subcategorization achieved by amalgamating diverse datasets and refining deep learning algorithms (138, 139). In the field of personalized therapy, AI is poised to expedite drug discovery processes and investigate combined treatment approaches (140). To facilitate disease surveillance and prognosis evaluation, AI can use wearable technologies and construct predictive models for continuous monitoring and risk assessment (105, 141). Furthermore, fostering interdisciplinary partnerships between medical and engineering sectors is imperative, particularly in the innovation of novel technologies such as digital twins (142). The establishment of extensive databases through data exchange and collaborative research initiatives across institutions will play a pivotal role in advancing autoimmune disease management (5).

### 4.4 Limitations

It should be noted that there are certain limitations to bibliometric analysis in practical implementation. Due to variations in inclusion criteria and sources among different databases, the publications covered may differ. However, mainstream analysis tools currently struggle to fully eliminate the impact of these differences. Therefore, the choice of using WoSCC as the primary database aims to ensure the credibility and reliability of the data to the greatest extent possible. Additionally, focusing solely on English literature may lead to some omissions, and alterations in institution names could also introduce biases. In summary, we acknowledge these potential issues in conducting bibliometric analysis and have referenced high-quality bibliometric literature in the research process to enhance the accuracy and reliability of the analysis results.

## 5 Conclusion

This study conducted a comprehensive bibliometric analysis on the application of AI in the field of autoimmune diseases, marking a pioneering endeavor. The analysis focused on publications from the past twenty years, aiming to thoroughly map the developmental trajectory and current status of this field. Through a systematic review of a large body of literature, we accurately identified key publications warranting attention in this field, meticulously examined the dynamic trends of key terms, pinpointed influential groups of authors, and highlighted professional journals and research institutions deserving close attention. Furthermore, this study vividly illustrated the evolution trajectory of research clusters in this field, providing a comprehensive overview of the current state, challenges, and future prospects of AI applications in the fields of autoimmune diseases. The findings serve as crucial reference points and direction indicators for further research and development in this field.

## Data Availability

The raw data supporting the conclusions of this article will be made available by the authors, without undue reservation.

## References

[1] Caliskan M Brown CD Maranville JC. A catalog of GWAS fine-mapping efforts in autoimmune disease. Am J Hum Genet. (2021) 108:549–63. doi: 10.1016/j.ajhg.2021.03.009 PMC8059376 33798443

[2] Gesheva V Szekeres Z Mihaylova N Dimitrova I Nikolova M Erdei A. Generation of gene-engineered chimeric DNA molecules for specific therapy of autoimmune diseases. Hum Gene Ther Methods. (2012) 23:357–65. doi: 10.1089/hgtb.2012.051 PMC4015069 23075110

[3] Moingeon P. Artificial intelligence-driven drug development against autoimmune diseases. Trends Pharmacol Sci. (2023) 44:411–24. doi: 10.1016/j.tips.2023.04.005 37268540

[4] Sadeghi P Karimi H Lavafian A Rashedi R Samieefar N Shafiekhani S. Machine learning and artificial intelligence within pediatric autoimmune diseases: applications, challenges, future perspective. Expert Rev Clin Immunol. (2024) 20:1219–36. doi: 10.1080/1744666X.2024.2359019 38771915

[5] Stafford IS Kellermann M Mossotto E Beattie RM MacArthur BD Ennis S. A systematic review of the applications of artificial intelligence and machine learning in autoimmune diseases. NPJ Digit Med. (2020) 3:30. doi: 10.1038/s41746-020-0229-3 32195365 PMC7062883

[6] Desvaux E Aussy A Hubert S Keime-Guibert F Blesius A Soret P. Model-based computational precision medicine to develop combination therapies for autoimmune diseases. Expert Rev Clin Immunol. (2022) 18:47–56. doi: 10.1080/1744666X.2022.2012452 34842494

[7] Katsila T Konstantinou E Lavda I Malakis H Papantoni I Skondra L. Pharmacometabolomics-aided pharmacogenomics in autoimmune disease. EBioMed. (2016) 5:40–5. doi: 10.1016/j.ebiom.2016.02.001 PMC4816847 27077110

[8] Laigle L Chadli L Moingeon P. Biomarker-driven development of new therapies for autoimmune diseases: current status and future promises. Expert Rev Clin Immunol. (2023) 19:305–14. doi: 10.1080/1744666X.2023.2172404 36680799

[9] Tong Y Cheng N Jiang X Wang K Wang F Lin X. The trends and hotspots in premature ovarian insufficiency therapy from 2000 to 2022. Int J Environ Res Public Health. (2022) 19:11728. doi: 10.3390/ijerph191811728 36142002 PMC9517308

[10] Zhang Q Li S Liu J Chen J. Global trends in nursing-related research on COVID-19: A bibliometric analysis. Front Public Health. (2022) 10:933555. doi: 10.3389/fpubh.2022.933555 35923953 PMC9339968

[11] Liu Y Huang H Zhou H Yuan Y Shi X. The evolution and future trends of stromal vascular fraction: A bibliometric analysis. Tissue Eng Part C Methods. (2024) 30:143–58. doi: 10.1089/ten.TEC.2023.0310 38205633

[12] Zhang XL Zheng Y Xia ML Wu YN Liu XJ Xie SK. Knowledge domain and emerging trends in vinegar research: A bibliometric review of the literature from WoSCC. Foods. (2020) 9:166. doi: 10.3390/foods9020166 32050682 PMC7074530

[13] Tao G Yang S Xu J Wang L Yang B. Global research trends and hotspots of artificial intelligence research in spinal cord neural injury and restoration-a bibliometrics and visualization analysis. Front Neurol. (2024) 15:1361235. doi: 10.3389/fneur.2024.1361235 38628700 PMC11018935

[14] Liu J Wang Y Zhang Q Wei J Zhou H. Scientometric analysis of public health emergencies: 1994-2020. Int J Environ Res Public Health. (2022) 19:640. doi: 10.3390/ijerph19020640 35055460 PMC8775579

[15] Liu YX Zhu C Wu ZX Lu LJ Yu YT. A bibliometric analysis of the application of artificial intelligence to advance individualized diagnosis and treatment of critical illness. Ann Transl Med. (2022) 10:854. doi: 10.21037/atm-22-913 36111047 PMC9469176

[16] Zhang XM Zhang X Luo X Guo HT Zhang LQ Guo JW. Knowledge mapping visualization analysis of the military health and medicine papers published in the web of science over the past 10 years. Mil Med Res. (2017) 4:23. doi: 10.1186/s40779-017-0131-8 28717517 PMC5508635

[17] Yao L Hui L Yang Z Chen X Xiao A. Freshwater microplastics pollution: Detecting and visualizing emerging trends based on Citespace II. Chemosphere. (2020) 245:125627. doi: 10.1016/j.chemosphere.2019.125627 31864046

[18] Martínez-Martínez C Esteve-Claramunt F Prieto-Callejero B Ramos-Pichardo JD. Stigma towards Mental Disorders among Nursing Students and Professionals: A Bibliometric Analysis. Int J Environ Res Public Health. (2022) 19:1839. doi: 10.3390/ijerph19031839 35162862 PMC8835101

[19] Peng C Kuang L Zhao J Ross AE Wang Z Ciolino JB. Bibliometric and visualized analysis of ocular drug delivery from 2001 to 2020. J Control Release. (2022) 345:625–45. doi: 10.1016/j.jconrel.2022.03.031 35321827

[20] Sweileh WM. Research trends on human trafficking: a bibliometric analysis using Scopus database. Global Health. (2018) 14:106. doi: 10.1186/s12992-018-0427-9 30409223 PMC6225706

[21] Zou M Zhang W Xu Y Zhu Y. Relationship between COPD and GERD: A bibliometrics analysis. Int J Chron Obstruct Pulmon Dis. (2022) 17:3045–59. doi: 10.2147/COPD.S391878 PMC9738194 36510485

[22] Rea F Marulli G Bortolotti L Feltracco P Zuin A Sartori F. Experience with the “da Vinci” robotic system for thymectomy in patients with myasthenia gravis: report of 33 cases. Ann Thorac Surg. (2006) 81:455–9. doi: 10.1016/j.athoracsur.2005.08.030 16427830

[23] Guan Y Zhang H Quang D Wang Z Parker S Pappas DA. Machine learning to predict anti-tumor necrosis factor drug responses of rheumatoid arthritis patients by integrating clinical and genetic markers. Arthritis Rheumatol. (2019) 71:1987–96. doi: 10.1002/art.41056 31342661

[24] Rückert JC Swierzy M Ismail M. Comparison of robotic and nonrobotic thoracoscopic thymectomy: a cohort study. J Thorac Cardiovasc Surg. (2011) 141:673–7. doi: 10.1016/j.jtcvs.2010.11.042 21335125

[25] Forbes JD Chen CY Knox NC Marrie RA El-Gabalawy H de Kievit T. A comparative study of the gut microbiota in immune-mediated inflammatory diseases-does a common dysbiosis exist. Microbiome. (2018) 6:221. doi: 10.1186/s40168-018-0603-4 30545401 PMC6292067

[26] Ritchie MD Denny JC Crawford DC Ramirez AH Weiner JB Pulley JM. Robust replication of genotype-phenotype associations across multiple diseases in an electronic medical record. Am J Hum Genet. (2010) 86:560–72. doi: 10.1016/j.ajhg.2010.03.003 PMC2850440 20362271

[27] Ford E Carroll JA Smith HE Scott D Cassell JA. Extracting information from the text of electronic medical records to improve case detection: a systematic review. J Am Med Inform Assoc. (2016) 23:1007–15. doi: 10.1093/jamia/ocv180 PMC4997034 26911811

[28] Ritchie ME Phipson B Wu D Hu Y Law CW Shi W. limma powers differential expression analyses for RNA-sequencing and microarray studies. Nucleic Acids Res. (2015) 43:e47. doi: 10.1093/nar/gkv007 25605792 PMC4402510

[29] Goldstein SD Yang SC. Assessment of robotic thymectomy using the Myasthenia Gravis Foundation of America Guidelines. Ann Thorac Surg. (2010) 89:1080–5; discussion 1085-6. doi: 10.1016/j.athoracsur.2010.01.038 20338310

[30] Marulli G Schiavon M Perissinotto E Bugana A Di Chiara F Rebusso A. Surgical and neurologic outcomes after robotic thymectomy in 100 consecutive patients with myasthenia gravis. J Thorac Cardiovasc Surg. (2013) 145:730–5; discussion 735-6. doi: 10.1016/j.jtcvs.2012.12.031 23312969

[31] Carroll RJ Thompson WK Eyler AE Mandelin AM Cai T Zink RM. Portability of an algorithm to identify rheumatoid arthritis in electronic health records. J Am Med Inform Assoc. (2012) 19:e162–9. doi: 10.1136/amiajnl-2011-000583 PMC3392871 22374935

[32] Liao KP Cai T Gainer V Goryachev S Zeng-treitler Q Raychaudhuri S. Electronic medical records for discovery research in rheumatoid arthritis. Arthritis Care Res (Hoboken). (2010) 62:1120–7. doi: 10.1002/acr.20184 PMC3121049 20235204

[33] Nanni L Lumini A Santos FLCD Paci M Hyttinen J. Ensembles of dense and dense sampling descriptors for the HEp-2 cells classification problem. Pattern Recog Lett. (2016) 82:28–35. doi: 10.1016/j.patrec.2016.01.026

[34] Bolyen E Rideout JR Dillon MR Bokulich NA Abnet CC Al-Ghalith GA. Reproducible, interactive, scalable and extensible microbiome data science using QIIME 2. Nat Biotechnol. (2019) 37:852–7. doi: 10.1038/s41587-019-0209-9 PMC7015180 31341288

[35] Vita R Overton JA Greenbaum JA Ponomarenko J Clark JD Cantrell JR. The immune epitope database (IEDB) 3.0. Nucleic Acids Res. (2015) 43:D405–12. doi: 10.1093/nar/gku938 PMC4384014 25300482

[36] Zhang X Zhang D Jia H Feng Q Wang D Liang D. The oral and gut microbiomes are perturbed in rheumatoid arthritis and partly normalized after treatment. Nat Med. (2015) 21:895–905. doi: 10.1038/nm.3914 26214836

[37] O’Sullivan KE Kreaden US Hebert AE Eaton D Redmond KC. A systematic review of robotic versus open and video assisted thoracoscopic surgery (VATS) approaches for thymectomy. Ann Cardiothorac Surg. (2019) 8:174–93. doi: 10.21037/acs.2019.02.04 PMC6462547 31032201

[38] Jamthikar AD Gupta D Puvvula A Johri AM Khanna NN Saba L. Cardiovascular risk assessment in patients with rheumatoid arthritis using carotid ultrasound B-mode imaging. Rheumatol Int. (2020) 40:1921–39. doi: 10.1007/s00296-020-04691-5 PMC7453675 32857281

[39] Danieli MG Brunetto S Gammeri L Palmeri D Claudi I Shoenfeld Y. Machine learning application in autoimmune diseases: State of art and future prospectives. Autoimmun Rev. (2024) 23:103496. doi: 10.1016/j.autrev.2023.103496 38081493

[40] Norgeot B Glicksberg BS Trupin L Lituiev D GianFrancesco M Oskotsky B. Assessment of a deep learning model based on electronic health record data to forecast clinical outcomes in patients with rheumatoid arthritis. JAMA Netw Open. (2019) 2:e190606. doi: 10.1001/jamanetworkopen.2019.0606 30874779 PMC6484652

[41] Jang S Kwon EJ Lee JJ. Rheumatoid arthritis: pathogenic roles of diverse immune cells. Int J Mol Sci. (2022) 23:905. doi: 10.3390/ijms23020905 35055087 PMC8780115

[42] Ayoub I Wolf BJ Geng L Song H Khatiwada A Tsao BP. Prediction models of treatment response in lupus nephritis. Kidney Int. (2022) 101:379–89. doi: 10.1016/j.kint.2021.11.014 PMC8792241 34871620

[43] Chen Y Huang S Chen T Liang D Yang J Zeng C. Machine learning for prediction and risk stratification of lupus nephritis renal flare. Am J Nephrol. (2021) 52:152–60. doi: 10.1159/000513566 33744876

[44] Jorge AM Smith D Wu Z Chowdhury T Costenbader K Zhang Y. Exploration of machine learning methods to predict systemic lupus erythematosus hospitalizations. Lupus. (2022) 31:1296–305. doi: 10.1177/09612033221114805 PMC9547899 35835534

[45] Aletaha D Smolen JS. Diagnosis and management of rheumatoid arthritis: A review. JAMA. (2018) 320:1360–72. doi: 10.1001/jama.2018.13103 30285183

[46] Smolen JS Landewé R Bijlsma J Burmester GR Dougados M Kerschbaumer A. EULAR recommendations for the management of rheumatoid arthritis with synthetic and biological disease-modifying antirheumatic drugs: 2019 update. Ann Rheum Dis. (2020) 79:685–99. doi: 10.1136/annrheumdis-2019-216655 31969328

[47] Yang K Hu Y Qi H. Digital health literacy: bibliometric analysis. J Med Internet Res. (2022) 24:e35816. doi: 10.2196/35816 35793141 PMC9301558

[48] Ciecierski-Holmes T Singh R Axt M Brenner S Barteit S. Artificial intelligence for strengthening healthcare systems in low- and middle-income countries: a systematic scoping review. NPJ Digit Med. (2022) 5:162. doi: 10.1038/s41746-022-00700-y 36307479 PMC9614192

[49] Rajpurkar P Lungren MP. The current and future state of AI interpretation of medical images. N Engl J Med. (2023) 388:1981–90. doi: 10.1056/NEJMra2301725 37224199

[50] Ueda D Matsumoto T Ehara S Yamamoto A Walston SL Ito A. Artificial intelligence-based model to classify cardiac functions from chest radiographs: a multi-institutional, retrospective model development and validation study. Lancet Digit Health. (2023) 5:e525–525e533. doi: 10.1016/S2589-7500(23)00107-3 37422342

[51] Collorone S Coll L Lorenzi M Lladó X Sastre-Garriga J Tintoré M. Artificial intelligence applied to MRI data to tackle key challenges in multiple sclerosis. Mult Scler. (2024) 30:767–84. doi: 10.1177/13524585241249422 38738527

[52] Momtazmanesh S Nowroozi A Rezaei N. Artificial intelligence in rheumatoid arthritis: current status and future perspectives: A state-of-the-art review. Rheumatol Ther. (2022) 9:1249–304. doi: 10.1007/s40744-022-00475-4 PMC9510088 35849321

[53] Chen W Huang Y Boyle B Lin S. The utility of including pathology reports in improving the computational identification of patients. J Pathol Inform. (2016) 7:46. doi: 10.4103/2153-3539.194838 27994938 PMC5139449

[54] Chen Y Carroll RJ Hinz ER Shah A Eyler AE Denny JC. Applying active learning to high-throughput phenotyping algorithms for electronic health records data. J Am Med Inform Assoc. (2013) 20:e253–9. doi: 10.1136/amiajnl-2013-001945 PMC3861916 23851443

[55] Lin C Karlson EW Dligach D Ramirez MP Miller TA Mo H. Automatic identification of methotrexate-induced liver toxicity in patients with rheumatoid arthritis from the electronic medical record. J Am Med Inform Assoc. (2015) 22:e151–61. doi: 10.1136/amiajnl-2014-002642 PMC5901122 25344930

[56] Ludvigsson JF Pathak J Murphy S Durski M Kirsch PS Chute CG. Use of computerized algorithm to identify individuals in need of testing for celiac disease. J Am Med Inform Assoc. (2013) 20:e306–10. doi: 10.1136/amiajnl-2013-001924 PMC3861918 23956016

[57] Murray SG Avati A Schmajuk G Yazdany J. Automated and flexible identification of complex disease: building a model for systemic lupus erythematosus using noisy labeling. J Am Med Inform Assoc. (2019) 26:61–5. doi: 10.1093/jamia/ocy154 PMC6308013 30476175

[58] Turner CA Jacobs AD Marques CK Oates JC Kamen DL Anderson PE. Word2Vec inversion and traditional text classifiers for phenotyping lupus. BMC Med Inform Decis Mak. (2017) 17:126. doi: 10.1186/s12911-017-0518-1 28830409 PMC5568290

[59] Zhou SM Fernandez-Gutierrez F Kennedy J Cooksey R Atkinson M Denaxas S. Defining disease phenotypes in primary care electronic health records by a machine learning approach: A case study in identifying rheumatoid arthritis. PloS One. (2016) 11:e0154515. doi: 10.1371/journal.pone.0154515 27135409 PMC4852928

[60] Khan A Shang N Petukhova L Zhang J Shen Y Hebbring SJ. Medical records-based genetic studies of the complement system. J Am Soc Nephrol. (2021) 32:2031–47. doi: 10.1681/ASN.2020091371 PMC8455263 33941608

[61] Ryu B Shin SY Baek RM Kim JW Heo E Kang I. Clinical genomic sequencing reports in electronic health record systems based on international standards: implementation study. J Med Internet Res. (2020) 22:e15040. doi: 10.2196/15040 32773368 PMC7445611

[62] Chaganti S Welty VF Taylor W Albert K Failla MD Cascio C. Discovering novel disease comorbidities using electronic medical records. PloS One. (2019) 14:e0225495. doi: 10.1371/journal.pone.0225495 31774837 PMC6880990

[63] Chen W Wei K Zhao W Zhou X. Estimation of key comorbidities for osteoarthritis progression based on the EMR-claims dataset. IEEE Access. (2019) 7:2431–42. doi: 10.1109/ACCESS.2019.2919998

[64] Escudié JB Rance B Malamut G Khater S Burgun A Cellier C. A novel data-driven workflow combining literature and electronic health records to estimate comorbidities burden for a specific disease: a case study on autoimmune comorbidities in patients with celiac disease. BMC Med Inform Decis Mak. (2017) 17:140. doi: 10.1186/s12911-017-0537-y 28962565 PMC5622531

[65] Kamps A Runhaar J de Ridder M de Wilde M van der Lei J Zhang W. Comorbidity in incident osteoarthritis cases and matched controls using electronic health record data. Arthritis Res Ther. (2023) 25:114. doi: 10.1186/s13075-023-03086-8 37403135 PMC10318652

[66] Widdifield J Ivers NM Bernatsky S Jaakkimainen L Bombardier C Thorne JC. Primary care screening and comorbidity management in rheumatoid arthritis in Ontario, Canada. Arthritis Care Res (Hoboken). (2017) 69:1495–503. doi: 10.1002/acr.23178 27998044

[67] Alessa M Alsugheir S Ahmed NAAAAE. Prevalence and predictors of seizure in patients with Alzheimer’s disease at a tertiary care center in Riyadh, Saudi Arabia. Trop J Pharm Res. (2021) 20:2381–6. doi: 10.4314/tjpr.v20i11.21

[68] Sharmeen S Nomani H Taub E Carlson H Yao Q. Polycystic ovary syndrome: epidemiologic assessment of prevalence of systemic rheumatic and autoimmune diseases. Clin Rheumatol. (2021) 40:4837–43. doi: 10.1007/s10067-021-05850-0 34216315

[69] Chamberlain AM Roger VL Noseworthy PA Chen LY Weston SA Jiang R. Identification of incident atrial fibrillation from electronic medical records. J Am Heart Assoc. (2022) 11:e023237. doi: 10.1161/JAHA.121.023237 35348008 PMC9075468

[70] Kapoor T Mahadeshwar P Hui-Yuen J Quinnies K Tatonetti N Gartshteyn Y. Prevalence of progressive multifocal leukoencephalopathy (PML) in adults and children with systemic lupus erythematosus. Lupus Sci Med. (2020) 7:e000388. doi: 10.1136/lupus-2020-000388 32513809 PMC7282388

[71] Kwa MC Ardalan K Laumann AE Nardone B West DP Silverberg JI. Validation of international classification of diseases codes for the epidemiologic study of dermatomyositis. Arthritis Care Res (Hoboken). (2017) 69:753–7. doi: 10.1002/acr.23010 27564726

[72] Chin CY Hsieh SY Tseng VS. eDRAM: Effective early disease risk assessment with matrix factorization on a large-scale medical database: A case study on rheumatoid arthritis. PloS One. (2018) 13:e0207579. doi: 10.1371/journal.pone.0207579 30475847 PMC6261027

[73] Daneshjou R Wang Y Bromberg Y Bovo S Martelli PL Babbi G. Working toward precision medicine: Predicting phenotypes from exomes in the Critical Assessment of Genome Interpretation (CAGI) challenges. Hum Mutat. (2017) 38:1182–92. doi: 10.1002/humu.23280 PMC5600620 28634997

[74] Liu C Ackerman HH Carulli JP. A genome-wide screen of gene-gene interactions for rheumatoid arthritis susceptibility. Hum Genet. (2011) 129:473–85. doi: 10.1007/s00439-010-0943-z 21210282

[75] Wei Z Wang W Bradfield J Li J Cardinale C Frackelton E. Large sample size, wide variant spectrum, and advanced machine-learning technique boost risk prediction for inflammatory bowel disease. Am J Hum Genet. (2013) 92:1008–12. doi: 10.1016/j.ajhg.2013.05.002 PMC3675261 23731541

[76] Noguchi K Saito I Namiki T Yoshimura Y Nakaguchi T. Reliability of non-contact tongue diagnosis for Sjögren’s syndrome using machine learning method. Sci Rep. (2023) 13:1334. doi: 10.1038/s41598-023-27764-4 36693892 PMC9872069

[77] Volkova A Ruggles KV. Predictive metagenomic analysis of autoimmune disease identifies robust autoimmunity and disease specific microbial signatures. Front Microbiol. (2021) 12:621310. doi: 10.3389/fmicb.2021.621310 33746917 PMC7969817

[78] Isakov O Dotan I Ben-Shachar S. Machine learning-based gene prioritization identifies novel candidate risk genes for inflammatory bowel disease. Inflammation Bowel Dis. (2017) 23:1516–23. doi: 10.1097/MIB.0000000000001222 28795970

[79] Shajari E Gagné D Malick M Roy P Noël JF Gagnon H. Application of SWATH mass spectrometry and machine learning in the diagnosis of inflammatory bowel disease based on the stool proteome. Biomedicines. (2024) 12:333. doi: 10.3390/biomedicines12020333 38397935 PMC10886680

[80] Alazwari A Abdollahian M Tafakori L Johnstone A Alshumrani RA Alhelal MT. Predicting age at onset of type 1 diabetes in children using regression, artificial neural network and Random Forest: A case study in Saudi Arabia. PloS One. (2022) 17:e0264118. doi: 10.1371/journal.pone.0264118 35226685 PMC8884498

[81] Nguyen C Varney MD Harrison LC Morahan G. Definition of high-risk type 1 diabetes HLA-DR and HLA-DQ types using only three single nucleotide polymorphisms. Diabetes. (2013) 62:2135–40. doi: 10.2337/db12-1398 PMC3661605 23378606

[82] Wei Z Wang K Qu HQ Zhang H Bradfield J Kim C. From disease association to risk assessment: an optimistic view from genome-wide association studies on type 1 diabetes. PloS Genet. (2009) 5:e1000678. doi: 10.1371/journal.pgen.1000678 19816555 PMC2748686

[83] Briggs FB Ramsay PP Madden E Norris JM Holers VM Mikuls TR. Supervised machine learning and logistic regression identifies novel epistatic risk factors with PTPN22 for rheumatoid arthritis. Genes Immun. (2010) 11:199–208. doi: 10.1038/gene.2009.110 20090771 PMC3118040

[84] González-Recio O de Maturana EL Vega AT Engelman CD Broman KW. Detecting single-nucleotide polymorphism by single-nucleotide polymorphism interactions in rheumatoid arthritis using a two-step approach with machine learning and a Bayesian threshold least absolute shrinkage and selection operator (LASSO) model. BMC Proc. (2009) 3 Suppl 7:S63. doi: 10.1186/1753-6561-3-s7-s63 20018057 PMC2795964

[85] Maksabedian Hernandez EJ Tingzon I Ampil L Tiu J. Identifying chronic disease patients using predictive algorithms in pharmacy administrative claims: an application in rheumatoid arthritis. J Med Econ. (2021) 24:1272–9. doi: 10.1080/13696998.2021.1999132 34704871

[86] Negi S Juyal G Senapati S Prasad P Gupta A Singh S. A genome-wide association study reveals ARL15, a novel non-HLA susceptibility gene for rheumatoid arthritis in North Indians. Arthritis Rheumatol. (2013) 65:3026–35. doi: 10.1002/art.38110 23918589

[87] Davis NA Lareau CA White BC Pandey A Wiley G Montgomery CG. Encore: Genetic Association Interaction Network centrality pipeline and application to SLE exome data. Genet Epidemiol. (2013) 37:614–21. doi: 10.1002/gepi.21739 PMC3955726 23740754

[88] Li H Zhou L Zhou W Zhang X Shang J Feng X. Decoding the mitochondrial connection: development and validation of biomarkers for classifying and treating systemic lupus erythematosus through bioinformatics and machine learning. BMC Rheumatol. (2023) 7:44. doi: 10.1186/s41927-023-00369-0 38044432 PMC10694981

[89] Barile B Ashtari P Stamile C Marzullo A Maes F Durand-Dubief F. Classification of multiple sclerosis clinical profiles using machine learning and grey matter connectome. Front Robot AI. (2022) 9:926255. doi: 10.3389/frobt.2022.926255 36313252 PMC9608344

[90] Mowry EM Hedström AK GianFrancesco MA Shao X Schaefer CA Shen L. Incorporating machine learning approaches to assess putative environmental risk factors for multiple sclerosis. Mult Scler Relat Disord. (2018) 24:135–41. doi: 10.1016/j.msard.2018.06.009 30005356

[91] Peng Y Zheng Y Tan Z Liu J Xiang Y Liu H. Prediction of unenhanced lesion evolution in multiple sclerosis using radiomics-based models: a machine learning approach. Mult Scler Relat Disord. (2021) 53:102989. doi: 10.1016/j.msard.2021.102989 34052741

[92] Amirkhani A Mosavi MR Mohammadi K Papageorgiou EI. A novel hybrid method based on fuzzy cognitive maps and fuzzy clustering algorithms for grading celiac disease. Neural Computing Appl. (2016) 30:1573–88. doi: 10.1007/s00521-016-2765-y

[93] George Y Aldeen M Garnavi R. Psoriasis image representation using patch-based dictionary learning for erythema severity scoring. Comput Med Imaging Graph. (2018) 66:44–55. doi: 10.1016/j.compmedimag.2018.02.004 29524784

[94] Lewis MJ. Predicting best treatment in rheumatoid arthritis. Semin Arthritis Rheumatol. (2024) 64S:152329. doi: 10.1016/j.semarthrit.2023.152329 38008706

[95] Lin C Karlson EW Canhao H Miller TA Dligach D Chen PJ. Automatic prediction of rheumatoid arthritis disease activity from the electronic medical records. PloS One. (2013) 8:e69932. doi: 10.1371/journal.pone.0069932 23976944 PMC3745469

[96] Niehaus KE Uhlig HH Clifton DA. Phenotypic characterisation of Crohn’s disease severity. Annu Int Conf IEEE Eng Med Biol Soc. (2015) 2015:7023–6. doi: 10.1109/EMBC.2015.7320009 26737909

[97] Raina A Hennessy R Rains M Allred J Hirshburg JM Diven DG. Objective measurement of erythema in psoriasis using digital color photography with color calibration. Skin Res Technol. (2016) 22:375–80. doi: 10.1111/srt.12276 PMC4851905 26517973

[98] Wang X Fu S Yu J Ma F Zhang L Wang J. Renal interferon-inducible protein 16 expression is associated with disease activity and prognosis in lupus nephritis. Arthritis Res Ther. (2023) 25:112. doi: 10.1186/s13075-023-03094-8 37393341 PMC10314472

[99] Salehi F Lopera Gonzalez LI Bayat S Kleyer A Zanca D Brost A. Machine learning prediction of treatment response to biological disease-modifying antirheumatic drugs in rheumatoid arthritis. J Clin Med. (2024) 13:3890. doi: 10.3390/jcm13133890 38999454 PMC11242607

[100] Tao W Concepcion AN Vianen M Marijnissen A Lafeber F Radstake T. Multiomics and machine learning accurately predict clinical response to adalimumab and etanercept therapy in patients with rheumatoid arthritis. Arthritis Rheumatol. (2021) 73:212–22. doi: 10.1002/art.41516 PMC7898388 32909363

[101] Ferrè L Clarelli F Pignolet B Mascia E Frasca M Santoro S. Combining clinical and genetic data to predict response to fingolimod treatment in relapsing remitting multiple sclerosis patients: A precision medicine approach. J Pers Med. (2023) 13:122. doi: 10.3390/jpm13010122 36675783 PMC9861774

[102] Lee S Kang S Eun Y Won HH Kim H Lee J. Machine learning-based prediction model for responses of bDMARDs in patients with rheumatoid arthritis and ankylosing spondylitis. Arthritis Res Ther. (2021) 23:254. doi: 10.1186/s13075-021-02635-3 34627335 PMC8501710

[103] Mease P Husni ME Kafka S Chakravarty SD Harrison DD Lo KH. Inhibition of radiographic progression across levels of composite index-defined disease activity in patients with active psoriatic arthritis treated with intravenous golimumab: results from a phase-3, double-blind, placebo-controlled trial. Arthritis Res Ther. (2020) 22:43. doi: 10.1186/s13075-020-2126-1 32143685 PMC7059340

[104] Nguyen NH Picetti D Dulai PS Jairath V Sandborn WJ Ohno-MaChado L. Machine learning-based prediction models for diagnosis and prognosis in inflammatory bowel diseases: A systematic review. J Crohns Colitis. (2022) 16:398–413. doi: 10.1093/ecco-jcc/jjab155 34492100 PMC8919806

[105] Toro-Domínguez D Martorell-Marugán J Martinez-Bueno M López-Domínguez R Carnero-Montoro E Barturen G. Scoring personalized molecular portraits identify Systemic Lupus Erythematosus subtypes and predict individualized drug responses, symptomatology and disease progression. Brief Bioinform. (2022) 23:bbac332. doi: 10.1093/bib/bbac332 35947992 PMC9487588

[106] Myasoedova E Athreya AP Crowson CS Davis JM Warrington KJ Walchak RC. Towards individualized prediction of response to methotrexate in early rheumatoid arthritis: a pharmacogenomics-driven machine learning approach. Arthritis Care Res (Hoboken). (2022) 74:879–88. doi: 10.1002/acr.24834 34902228

[107] Alaqtash M Sarkodie-Gyan T Yu H Fuentes O Brower R Abdelgawad A. Automatic classification of pathological gait patterns using ground reaction forces and machine learning algorithms. Annu Int Conf IEEE Eng Med Biol Soc. (2011) 2011:453–7. doi: 10.1109/IEMBS.2011.6090063 22254346

[108] de Seny D Fillet M Meuwis MA Geurts P Lutteri L Ribbens C. Discovery of new rheumatoid arthritis biomarkers using the surface-enhanced laser desorption/ionization time-of-flight mass spectrometry ProteinChip approach. Arthritis Rheumatol. (2005) 52:3801–12. doi: 10.1002/art.21607 16320331

[109] Liu C Pan C Shen J Wang H Yong L. MALDI-TOF MS combined with magnetic beads for detecting serum protein biomarkers and establishment of boosting decision tree model for diagnosis of colorectal cancer. Int J Med Sci. (2011) 8:39–47. doi: 10.7150/ijms.8.39 21234268 PMC3020391

[110] Niu Q Huang Z Shi Y Wang L Pan X Hu C. Specific serum protein biomarkers of rheumatoid arthritis detected by MALDI-TOF-MS combined with magnetic beads. Int Immunol. (2010) 22:611–8. doi: 10.1093/intimm/dxq043 20497952

[111] Arasaradnam RP Westenbrink E McFarlane MJ Harbord R Chambers S O’Connell N. Differentiating coeliac disease from irritable bowel syndrome by urinary volatile organic compound analysis–a pilot study. PloS One. (2014) 9:e107312. doi: 10.1371/journal.pone.0107312 25330367 PMC4199520

[112] Cowen EW Liu CW Steinberg SM Kang S Vonderheid EC Kwak HS. Differentiation of tumour-stage mycosis fungoides, psoriasis vulgaris and normal controls in a pilot study using serum proteomic analysis. Br J Dermatol. (2007) 157:946–53. doi: 10.1111/j.1365-2133.2007.08185.x 17854367

[113] Ohanian D Brown A Sunnquist M Furst J Nicholson L Klebek L. Identifying key symptoms differentiating myalgic encephalomyelitis and chronic fatigue syndrome from multiple sclerosis. Neurol (ECronicon). (2016) 4:41–5. PMC5214344 28066845

[114] Liu J Chen N. A 9 mRNAs-based diagnostic signature for rheumatoid arthritis by integrating bioinformatic analysis and machine-learning. J Orthop Surg Res. (2021) 16:44. doi: 10.1186/s13018-020-02180-w 33430905 PMC7802293

[115] Saccà V Sarica A Novellino F Barone S Tallarico T Filippelli E. Evaluation of machine learning algorithms performance for the prediction of early multiple sclerosis from resting-state FMRI connectivity data. Brain Imaging Behav. (2019) 13:1103–14. doi: 10.1007/s11682-018-9926-9 29992392

[116] Lopez C Tucker S Salameh T Tucker C. An unsupervised machine learning method for discovering patient clusters based on genetic signatures. J BioMed Inform. (2018) 85:30–9. doi: 10.1016/j.jbi.2018.07.004 PMC6621561 30016722

[117] Mossotto E Ashton JJ Coelho T Beattie RM MacArthur BD Ennis S. Classification of paediatric inflammatory bowel disease using machine learning. Sci Rep. (2017) 7:2427. doi: 10.1038/s41598-017-02606-2 28546534 PMC5445076

[118] Orange DE Agius P DiCarlo EF Robine N Geiger H Szymonifka J. Identification of three rheumatoid arthritis disease subtypes by machine learning integration of synovial histologic features and RNA sequencing data. Arthritis Rheumatol. (2018) 70:690–701. doi: 10.1002/art.40428 29468833 PMC6336443

[119] Wang Y Wei W Ouyang R Chen R Wang T Yuan X. Novel multiclass classification machine learning approach for the early-stage classification of systemic autoimmune rheumatic diseases. Lupus Sci Med. (2024) 11:e001125. doi: 10.1136/lupus-2023-001125 38302133 PMC10831448

[120] Georga EI Protopappas VC Ardigò D Polyzos D Fotiadis DI. A glucose model based on support vector regression for the prediction of hypoglycemic events under free-living conditions. Diabetes Technol Ther. (2013) 15:634–43. doi: 10.1089/dia.2012.0285 23848178

[121] Georga EI Protopappas VC Polyzos D Fotiadis DI. Evaluation of short-term predictors of glucose concentration in type 1 diabetes combining feature ranking with regression models. Med Biol Eng Comput. (2015) 53:1305–18. doi: 10.1007/s11517-015-1263-1 25773366

[122] Arumalla N Chan C Gibson M Man YL Adas MA Norton S. The clinical impact of electronic patient-reported outcome measures in the remote monitoring of inflammatory arthritis: A systematic review and meta-analysis. Arthritis Rheumatol. (2023) 75:1892–903. doi: 10.1002/art.42559 37204273

[123] Barnett M Wang D Beadnall H Bischof A Brunacci D Butzkueven H. A real-world clinical validation for AI-based MRI monitoring in multiple sclerosis. NPJ Digit Med. (2023) 6:196. doi: 10.1038/s41746-023-00940-6 37857813 PMC10587188

[124] Majidova K Handfield J Kafi K Martin RD Kubinski R. Role of digital health and artificial intelligence in inflammatory bowel disease: A scoping review. Genes (Basel). (2021) 12:1465. doi: 10.3390/genes12101465 34680860 PMC8535572

[125] McGinnis RS Mahadevan N Moon Y Seagers K Sheth N Wright JA Jr. A machine learning approach for gait speed estimation using skin-mounted wearable sensors: From healthy controls to individuals with multiple sclerosis. PloS One. (2017) 12:e0178366. doi: 10.1371/journal.pone.0178366 28570570 PMC5453431

[126] Guthridge JM Lu R Tran LT Arriens C Aberle T Kamp S. Adults with systemic lupus exhibit distinct molecular phenotypes in a cross-sectional study. EClinicalMed. (2020) 20:100291. doi: 10.1016/j.eclinm.2020.100291 PMC7058913 32154507

[127] Smith MA Chiang CC Zerrouki K Rahman S White WI Streicher K. Using the circulating proteome to assess type I interferon activity in systemic lupus erythematosus. Sci Rep. (2020) 10:4462. doi: 10.1038/s41598-020-60563-9 32157125 PMC7064569

[128] Yaung KN Yeo JG Kumar P Wasser M Chew M Ravelli A. Artificial intelligence and high-dimensional technologies in the theragnosis of systemic lupus erythematosus. Lancet Rheumatol. (2023) 5:e151–151e165. doi: 10.1016/S2665-9913(23)00010-3 38251610

[129] Aterido A Cañete JD Tornero J Blanco F Fernández-Gutierrez B Pérez C. A combined transcriptomic and genomic analysis identifies a gene signature associated with the response to anti-TNF therapy in rheumatoid arthritis. Front Immunol. (2019) 10:1459. doi: 10.3389/fimmu.2019.01459 31312201 PMC6614444

[130] Robert M Miossec P. IL-17 in rheumatoid arthritis and precision medicine: from synovitis expression to circulating bioactive levels. Front Med (Lausanne). (2018) 5:364. doi: 10.3389/fmed.2018.00364 30693283 PMC6339915

[131] Yoosuf N Maciejewski M Ziemek D Jelinsky SA Folkersen L Müller M. Early prediction of clinical response to anti-TNF treatment using multi-omics and machine learning in rheumatoid arthritis. Rheumatol (Oxford). (2022) 61:1680–9. doi: 10.1093/rheumatology/keab521 PMC8996791 34175943

[132] Zhao J Guo S Schrodi SJ He D. Molecular and cellular heterogeneity in rheumatoid arthritis: mechanisms and clinical implications. Front Immunol. (2021) 12:790122. doi: 10.3389/fimmu.2021.790122 34899757 PMC8660630

[133] Fasano S Milone A Nicoletti GF Isenberg DA Ciccia F. Precision medicine in systemic lupus erythematosus. Nat Rev Rheumatol. (2023) 19:331–42. doi: 10.1038/s41584-023-00948-y 37041269

[134] Guthridge JM Wagner CA James JA. The promise of precision medicine in rheumatology. Nat Med. (2022) 28:1363–71. doi: 10.1038/s41591-022-01880-6 PMC9513842 35788174

[135] Kelly CJ Karthikesalingam A Suleyman M Corrado G King D. Key challenges for delivering clinical impact with artificial intelligence. BMC Med. (2019) 17:195. doi: 10.1186/s12916-019-1426-2 31665002 PMC6821018

[136] Obermeyer Z Emanuel EJ. Predicting the future - big data, machine learning, and clinical medicine. N Engl J Med. (2016) 375:1216–9. doi: 10.1056/NEJMp1606181 PMC5070532 27682033

[137] Toro-Domínguez D Alarcón-Riquelme ME. Precision medicine in autoimmune diseases: fact or fiction. Rheumatol (Oxford). (2021) 60:3977–85. doi: 10.1093/rheumatology/keab448 34003926

[138] Anchang CG Xu C Raimondo MG Atreya R Maier A Schett G. The potential of OMICs technologies for the treatment of immune-mediated inflammatory diseases. Int J Mol Sci. (2021) 22:7506. doi: 10.3390/ijms22147506 34299122 PMC8306614

[139] Martorell-Marugán J López-Domínguez R García-Moreno A Toro-Domínguez D Villatoro-García JA Barturen G. A comprehensive database for integrated analysis of omics data in autoimmune diseases. BMC Bioinf. (2021) 22:343. doi: 10.1186/s12859-021-04268-4 PMC8223391 34167460

[140] Vamathevan J Clark D Czodrowski P Dunham I Ferran E Lee G. Applications of machine learning in drug discovery and development. Nat Rev Drug Discovery. (2019) 18:463–77. doi: 10.1038/s41573-019-0024-5 PMC6552674 30976107

[141] Burmester GR. Rheumatology 4.0: big data, wearables and diagnosis by computer. Ann Rheum Dis. (2018) 77:963–5. doi: 10.1136/annrheumdis-2017-212888 PMC6029631 29802224

[142] Kamel Boulos MN Zhang P. Digital twins: from personalised medicine to precision public health. J Pers Med. (2021) 11:745. doi: 10.3390/jpm11080745 34442389 PMC8401029

