# Supplementary material for: Artificial intelligence in autoimmune diseases: a bibliometric exploration of the past two decades
Source: Front Immunol. 2025 Apr 22;16:1525462. doi: 10.3389/fimmu.2025.1525462 (PMC12052778; doi:10.3389/fimmu.2025.1525462)
Supplement: Supplementary file 1 [file Table1.docx]

**Table S1.** Retrieval strategy of publications in the study

| Retrieval  term | Value |
| --- | --- |
| Citation index | Web of Science Core Collection (WoSCC); Science Citation Index Expanded (SCI-EXPANDED) |
| Topic | #1.TS= (“autoimmune diseases*” OR “autoimmune*” OR “anti-glomerular basement membrane disease*” OR “anti-neutrophil cytoplasmic antibody-associated vasculitis*” OR “Churg-Strauss syndrome*” OR “granulomatosis with polyangiitis*” OR “microscopic polyangiitis*” OR “antiphospholipid syndrome*” OR “rheumatoid arthritis*” OR “rheumatoid nodule*” OR “rheumatoid vasculitis*” OR “Felty syndrome*”OR “Sjogren's Syndrome*” OR “Still's disease*” OR “autoimmune diseases of the nervous system*” OR “polyradiculoneuropathy*” OR “demyelinating autoimmune diseases*” OR “Stiff-Person syndrome*” OR ”myasthenia gravis*” OR "systemic lupus erythematosus*" OR "lupus nephritis*" OR "lupus vasculitis*" OR lupus* OR pemphigus* OR "undifferentiated connective tissue diseases*" OR "systemic scleroderma*")  #2.TS= (“Artificial Intelligence” OR “Robot*” OR “Natural Language Processing” OR “Deep Learn*” OR “Machine Learn*” OR “Machine intelligence” OR “Hierarchical Learn*” OR “Autonomous System” OR “Intelligent System” OR “Artificial neural network*” OR “thinking computer system” OR “evolutionary computation” OR “hybrid intelligent system”) |
| Document  type | Article OR Review Article |
| Language | English |
| Timespan | 2003.01.01-2024.07.01 |
