# Supplementary material for: Artificial intelligence in autoimmune diseases: a bibliometric exploration of the past two decades
Source: Front Immunol. 2025 Apr 22;16:1525462. doi: 10.3389/fimmu.2025.1525462 (PMC12052778; doi:10.3389/fimmu.2025.1525462)
Supplement: Supplementary file 2 [file Table2.docx]

**Table S2.** Basic information on the distribution of the publications

| **Categories** | **Publications** | **Research articles** | **Review articles** | **Authors** | **institutions** | **Journals** |
| --- | --- | --- | --- | --- | --- | --- |
| Amount | 1,695 | 1,409 | 286 | 10,915 | 7,070 | 703 |
