# Supplementary material for: Artificial intelligence in autoimmune diseases: a bibliometric exploration of the past two decades
Source: Front Immunol. 2025 Apr 22;16:1525462. doi: 10.3389/fimmu.2025.1525462 (PMC12052778; doi:10.3389/fimmu.2025.1525462)
Supplement: Supplementary file 3 [file Table3.docx]

**Table S3.** The information of the top 30 literature sorted by LCS score

| **NO.** | **Article information** | **Journal** | **LCS** | **GCS** |
| --- | --- | --- | --- | --- |
| 1 | Experience with the "da Vinci" robotic system for thymectomy in patients with myasthenia gravis: report of 33 cases | Annals of thoracic surgery | 45 | 104 |
| 2 | Comparison of robotic and nonrobotic thoracoscopic thymectomy: A cohort study | Journal of thoracic and cardiovascular surgery | 38 | 97 |
| 3 | Machine Learning to Predict Anti-Tumor Necrosis Factor Drug Responses of Rheumatoid Arthritis Patients by Integrating Clinical and Genetic Markers | Arthritis & rheumatology | 34 | 75 |
| 4 | Early experience with robot-assisted surgery for mediastinal masses | Annals of thoracic surgery | 32 | 109 |
| 5 | Thoracoscopic thymectomy with the da Vinci robotic system for myasthenia gravis | Annals of the new York academy of sciences | 32 | 76 |
| 6 | Surgical and neurologic outcomes after robotic thymectomy in 100 consecutive patients with myasthenia gravis | Journal of thoracic and cardiovascular surgery | 32 | 69 |
| 7 | A comparison of outcomes after robotic open extended thymectomy for myasthenia gravis | European journal of cardio-thoracic surgery | 31 | 56 |
| 8 | Portability of an algorithm to identify rheumatoid arthritis in electronic health records | Journal of the American medical informatics association | 31 | 157 |
| 9 | Application of robotic-assisted techniques to the surgical evaluation and treatment of the anterior mediastinum | Annals of thoracic surgery | 27 | 86 |
| 10 | Assessment of robotic thymectomy using the Myasthenia Gravis Foundation of America Guidelines | Annals of thoracic surgery | 27 | 46 |
| 11 | Identification of Three Rheumatoid Arthritis Disease Subtypes by Machine Learning Integration of Synovial Histologic Features and RNA Sequencing Data | Arthritis & rheumatology | 25 | 127 |
| 12 | Video-assisted thoracoscopic surgery versus robotic-assisted thoracoscopic surgery thymectomy | Annals of thoracic surgery | 24 | 69 |
| 13 | Long-term follow-up after robotic thymectomy for nonthymomatous myasthenia gravis | Annals of thoracic surgery | 24 | 39 |
| 14 | Robot-aided thoracoscopic thymectomy for early-stage thymoma: a multicenter European study | Journal of thoracic and cardiovascular surgery | 23 | 86 |
| 15 | Lupus or not? SLE Risk Probability Index (SLERPI): a simple, clinician-friendly machine learning-based model to assist the diagnosis of systemic lupus erythematosus | Annals of the rheumatic diseases | 22 | 45 |
| 16 | Robotic extended thymectomy for early-stage thymomas | European journal of cardio-thoracic surgery | 21 | 39 |
| 17 | Detection of rheumatoid arthritis from hand radiographs using a convolutional neural network | Clinical rheumatology | 21 | 45 |
| 18 | Robot-assisted thymectomy is superior to transsternal thymectomy | Surgical endoscopy and other interventional techniques | 19 | 56 |
| 19 | Early clinical outcomes of robot-assisted surgery for anterior mediastinal mass: its superiority over a conventional sternotomy approach evaluated by propensity score matching | European journal of cardio-thoracic surgery. | 19 | 59 |
| 20 | Multiomics and Machine Learning Accurately Predict Clinical Response to Adalimumab and Etanercept Therapy in Patients With Rheumatoid Arthritis | Arthritis & rheumatology | 19 | 81 |
| 21 | Identifying lupus patients in electronic health records: Development and validation of machine learning algorithms and application of rule-based algorithms | Seminars in arthritis and rheumatism | 17 | 47 |
| 22 | An introduction to machine learning and analysis of its use in rheumatic diseases | Nature reviews rheumatology | 17 | 32 |
| 23 | Robotic thymectomy in patients with myasthenia gravis: neurological and surgical outcomes | European journal of cardio-thoracic surgery | 16 | 40 |
| 24 | Multi-institutional European experience of robotic thymectomy for thymoma | Annals of cardiothoracic surgery | 16 | 46 |
| 25 | Automatic localization of anatomical regions in medical ultrasound images of rheumatoid arthritis using deep learning | Proceedings of the institution of mechanical engineers part h-journal of engineering in medicine | 16 | 21 |
| 26 | Bone erosion scoring for rheumatoid arthritis with deep convolutional neural networks | Computers & electrical engineering | 16 | 25 |
| 27 | Detection of Flares by Decrease in Physical Activity, Collected Using Wearable Activity Trackers in Rheumatoid Arthritis or Axial Spondyloarthritis: An Application of Machine Learning Analyses in Rheumatology | Arthritis care & research | 15 | 80 |
| 28 | Ten-year experience of mediastinal robotic surgery in a single referral centre | European journal of cardio-thoracic surgery | 13 | 44 |
| 29 | 8 years' experience with robotic thymectomy for thymomas | Surgical endoscopy and other interventional techniques | 13 | 29 |
| 30 | Disease-associated and patient-specific immune cell signatures in juvenile-onset systemic lupus erythematosus: patient stratification using a machine-learning approach | Lancet rheumatology | 13 | 43 |

LCS: The total local citation score; GCS: The total global citation score
