# Supplementary material for: Artificial intelligence in autoimmune diseases: a bibliometric exploration of the past two decades
Source: Front Immunol. 2025 Apr 22;16:1525462. doi: 10.3389/fimmu.2025.1525462 (PMC12052778; doi:10.3389/fimmu.2025.1525462)
Supplement: Supplementary file 4 [file Table4.docx]

**Table S4.** Summary of the largest 9 clusters

| **Avg. Yr.** | **Cluster** | **Size** | **S** |
| --- | --- | --- | --- |
| 2010 | #1 Robotic Thoracic Surgery | 83 | 0.931 |
| 2011 | #2 Computable Representation | 52 | 0.99 |
| 2015 | #4 Hep-2 Cells Classification Problem | 37 | 0.991 |
| 2015 | #5 Human Health | 27 | 1 |
| 2016 | #8 Thoracoscopic Surgery | 21 | 0.983 |
| 2017 | #6 Using Carotid Ultrasound | 26 | 0.975 |
| 2019 | #0 Artificial Intelligence | 126 | 0.862 |
| 2019 | #3 Automated Detection | 39 | 0.951 |
| 2022 | #7 Using Genomic Data | 23 | 0.979 |

S: Silhouette; Avg. Yr.: Average Year
